# The Video Head Impulse Test

**DOI:** 10.3389/fneur.2017.00258

**Published:** 2017-06-09

**Authors:** G. M. Halmagyi, Luke Chen, Hamish G. MacDougall, Konrad P. Weber, Leigh A. McGarvie, Ian S. Curthoys

**Affiliations:** ^1^Neurology Department, Institute of Clinical Neurosciences, Royal Prince Alfred Hospital, Camperdown, NSW, Australia; ^2^Vestibular Research Laboratory, School of Psychology, The University of Sydney, Sydney, NSW, Australia; ^3^Department of Ophthalmology, University Hospital Zurich, University of Zurich, Zurich, Switzerland; ^4^Department of Neurology, University Hospital Zurich, University of Zurich, Zurich, Switzerland

**Keywords:** vestibular, vestibulo-ocular reflex, VOR, semicircular canal, video head impulse test, head impulse test, SHIMP

## Abstract

In 1988, we introduced impulsive testing of semicircular canal (SCC) function measured with scleral search coils and showed that it could accurately and reliably detect impaired function even of a single lateral canal. Later we showed that it was also possible to test individual vertical canal function in peripheral and also in central vestibular disorders and proposed a physiological mechanism for why this might be so. For the next 20 years, between 1988 and 2008, impulsive testing of individual SCC function could only be accurately done by a few aficionados with the time and money to support scleral search-coil systems—an expensive, complicated and cumbersome, semi-invasive technique that never made the transition from the research lab to the dizzy clinic. Then, in 2009 and 2013, we introduced a video method of testing function of each of the six canals individually. Since 2009, the method has been taken up by most dizzy clinics around the world, with now close to 100 refereed articles in PubMed. In many dizzy clinics around the world, video Head Impulse Testing has supplanted caloric testing as the initial and in some cases the final test of choice in patients with suspected vestibular disorders. Here, we consider seven current, interesting, and controversial aspects of video Head Impulse Testing: (1) introduction to the test; (2) the progress from the head impulse protocol (HIMPs) to the new variant—suppression head impulse protocol (SHIMPs); (3) the physiological basis for head impulse testing; (4) practical aspects and potential pitfalls of video head impulse testing; (5) problems of vestibulo-ocular reflex gain calculations; (6) head impulse testing in central vestibular disorders; and (7) to stay right up-to-date—new clinical disease patterns emerging from video head impulse testing. With thanks and appreciation we dedicate this article to our friend, colleague, and mentor, Dr Bernard Cohen of Mount Sinai Medical School, New York, who since his first article 55 years ago on compensatory eye movements induced by vertical SCC stimulation has become one of the giants of the vestibular world.

## Introduction

In 1984, we tested a patient with bilateral vestibular schwannomas who had had both vestibular nerves surgically sectioned (1). We measured the horizontal smooth compensatory eye movements in response to passive, low-frequency (0.2 Hz), low-acceleration, sinusoidal horizontal rotations, first while the patient stared at an earth-fixed LED target in an otherwise totally dark room, and then, while he imagined the target after it had been switched off. Even without any vestibular sensory input, this patient could nonetheless generate reasonably smooth compensatory eye movement responses to that stimulus, which shows that low-frequency, low-acceleration head rotation is not a valid indicator of semicircular canal (SCC) function. In response to this stimulus, other oculomotor control mechanisms can generate the compensatory eye movements.

However, when staring at the earth-fixed target during small, fast, passive unpredictable horizontal head turns (~15°, 100°/s, 1,000°/s^2^), for the first 100 ms or so, he could not generate any eye movement response to keep his gaze on target so that his gaze went with his head. Eye movement responses to small, brief, fast, unpredictable horizontal head turns (head impulses) are, in contrast to passive, slow, predictable head turns, a valid indicator of SCC function. The angular acceleration of a head impulse—up to 4,000°/s^2^—is what occurs during normal head movements. A head impulse is a purely vestibular test because it is too fast for other oculomotor control systems (such as smooth pursuit, optokinetic, cervico-ocular reflex). The head impulse test (HIT) can be done in a normally lit room—visual stimuli do not play a role in generating the response, which means this purely vestibular test can be done anywhere—a dark room is not necessary so long as the head turn is fast—above 150°/s, passive and unpredictable.

## From Head Impulses to SHIMPs

### The Standard HIT

**Figure 1:**
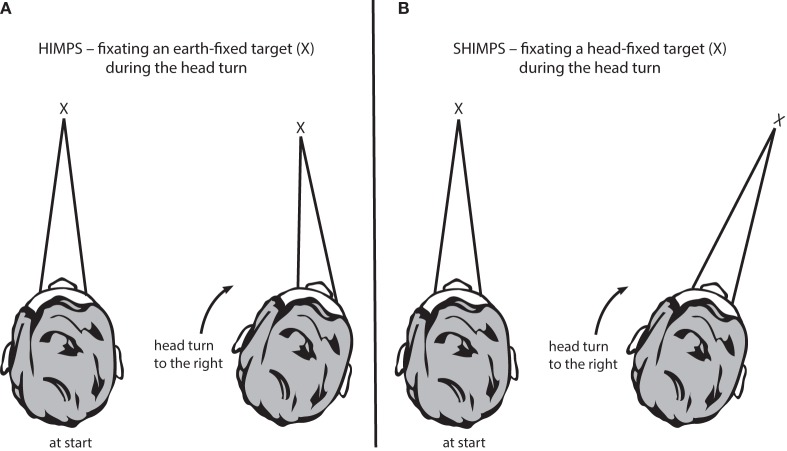
The two test protocols for testing semicircular canal function. **(A)** In the HIMPs protocol (the classical test protocol), the person is required to maintain fixation on an earth-fixed target during a small, unpredictable, passive, head turn. Healthy subjects will not make any saccades at all or only small saccades. **(B)** In the SHIMPs protocol, the head turn is identical but the instructions are different—the person must maintain fixation on a head-fixed target (a spot from a head-mounted laser projected onto the wall in front of the subject). Healthy subjects make large saccades (see text for explanation).

**Figure 2:**
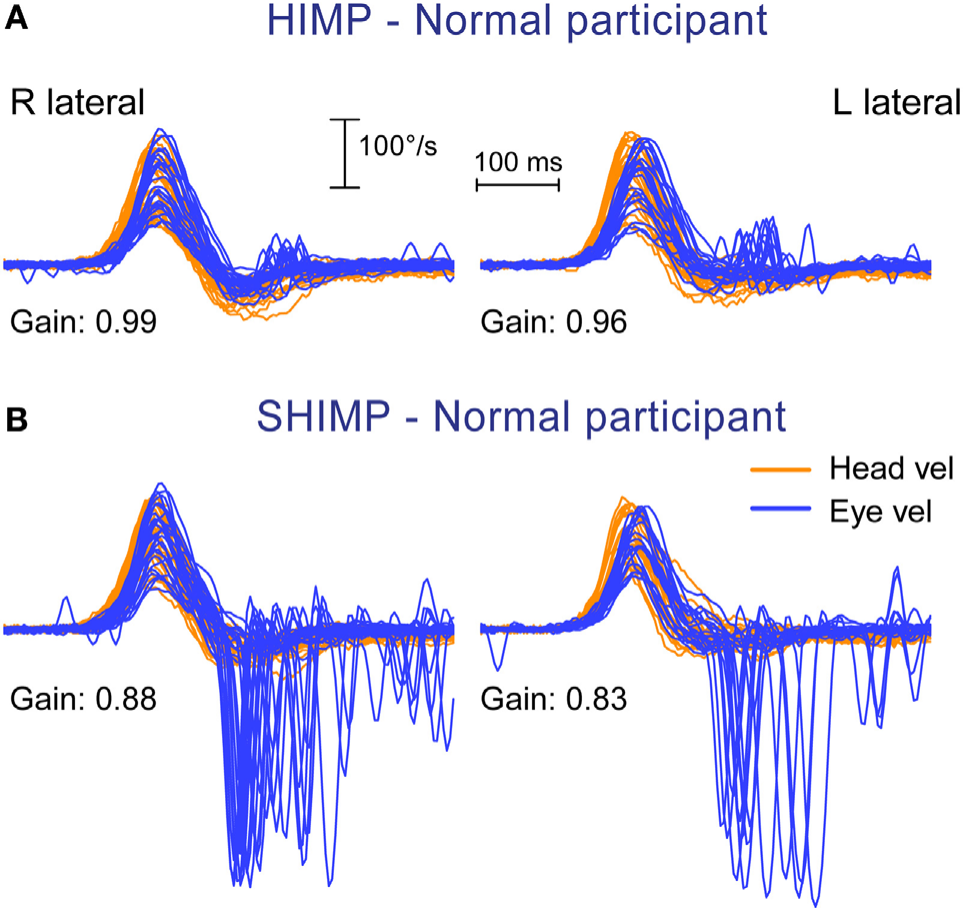
Superimposed head and eye velocity records for a healthy subject during HIMPs trials **(A)** and SHIMPs trials **(B)**. **(A)** During conventional HIMP trials, a typical healthy control elicits only very small mostly positive (compensatory) catch-up saccades after the end of the head impulse. **(B)** During SHIMP trials, the same healthy control shows large negative (anticompensatory) saccades after the end of the head impulse reflecting anticompensatory eye movements back to the head-fixed target. Both protocols give similar but slightly lower vestibulo-ocular reflex gain values during SHIMP trials compared to HIMP trials, but the saccade pattern is exactly complementary. Head velocity, orange traces; inverted eye velocity, blue traces; HIMP, conventional head impulse protocol; SHIMP, suppression head impulse protocol.

**Figure 3:**
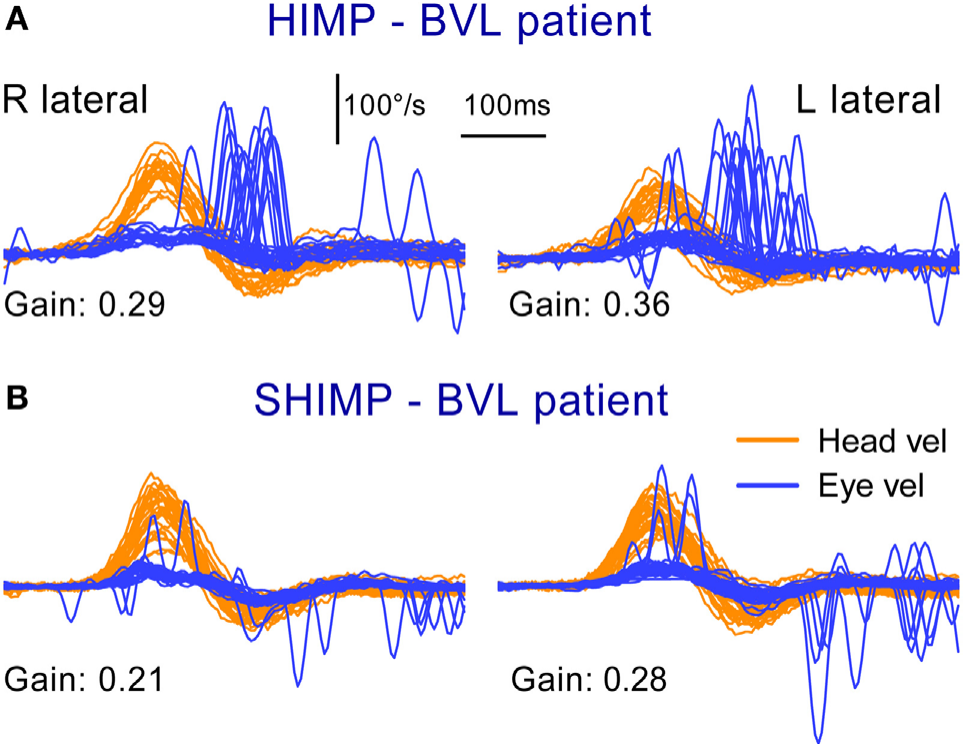
Superimposed head and eye velocity records for a patient with BVL during HIMPs trials **(A)** and SHIMPs trials **(B)**. The results for a typical patient with complete BVL showing a reversed saccadic pattern during HIMP and SHIMP compared to a healthy control (Figure 2). **(A)** During standard HIMP trials, the patient with BVL elicits mostly overt positive (compensatory) catch-up saccades after the head impulse. **(B)** During SHIMP, the same patient with BVL shows only very few downward (anticompensatory) saccades after the end of the head impulse back to the head-fixed target. Both protocols give similar but slightly lower vestibulo-ocular reflex gain values during SHIMP compared to HIMP, but there is a complementary saccade pattern, which is reversed compared to healthy controls. Head velocity, orange traces; inverted eye velocity, blue traces. BVL, bilateral vestibular loss; HIMP, conventional head impulse protocol; SHIMP, suppression head impulse protocol. Reproduced with permission of Wolters Kluwer from the study by MacDougall et al. (3); http://www.neurology.org.

In the HIT, the clinician turns the patient’s head abruptly and unpredictably in the plane of a SCC pair, about 15° in about 100 ms, and observes the instantaneous compensatory eye movement response (Figure 1A). During each head impulse, the eye movement response of a healthy subject will compensate for head turn and gaze will stay fixed on the earth-fixed fixation target (Figure 2A); however, the eyes of a patient without vestibular function (an “avestibular” patient) will move with the head so that the patient has to make a corrective saccade at the end of each head impulse in order to return his gaze to the earth-fixed target (Figure 3A). This “overt” corrective or catch-up saccade observed by the clinician is the clinical sign of canal paresis (2). The contrast is that during the head turn subjects with normal SCC function make smooth compensatory eye movements, which keep gaze on the earth-fixed target, and do not need to make catch-up saccades. The overt catch-up saccades are useful because clinicians can observe them even in bedridden patients. But of course the major drawback of any clinical sign of canal paresis is that it is subjective—there is no objective verifiable record of exactly what the patient did.

**Figure 4:**
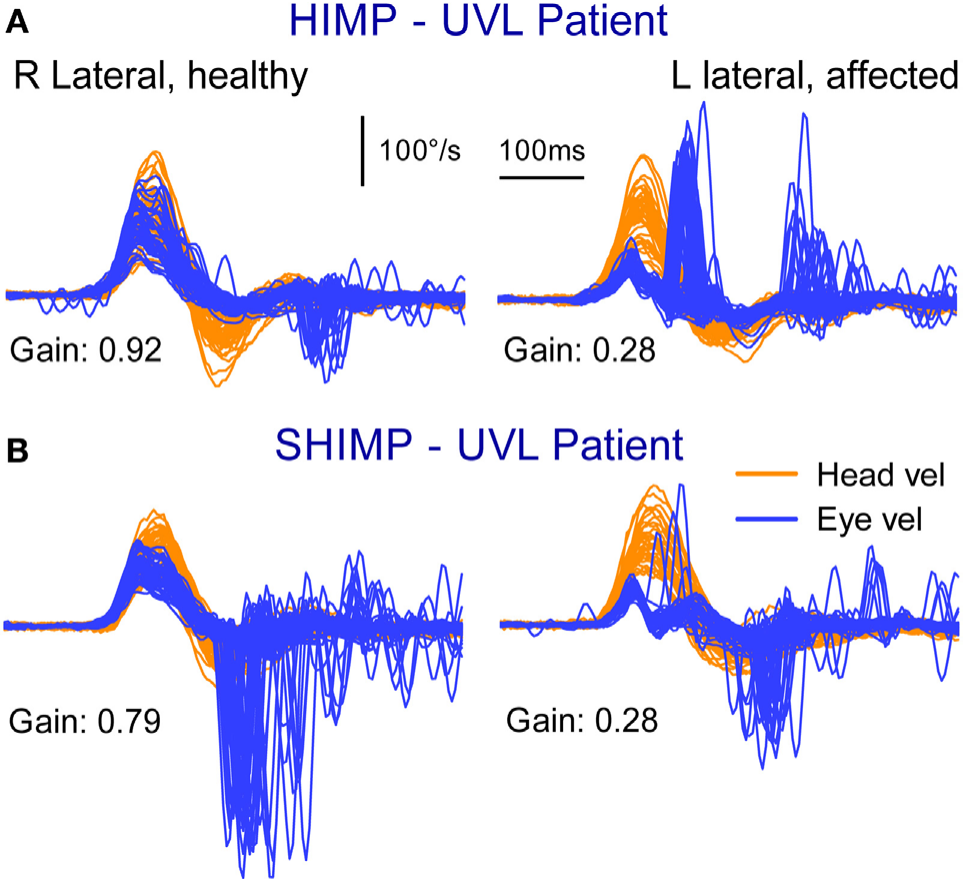
Superimposed head and eye velocity records for a patient with unilateral vestibular loss (UVL) during HIMPs trials **(A)** and SHIMPs trials **(B)**. **(A)** For rotations to their healthy (right) side, the patient shows the usual HIMPs pattern of slow compensatory eye movement with few saccades. During rotations to the affected (left) side, there is a reduced slow-phase eye velocity and covert and overt saccades. **(B)** For rotations to their affected side, there are few SHIMPs saccades, whereas for rotations to their healthy left side, there are many large SHIMP saccades. Reproduced with permission of Wolters Kluwer from the study by MacDougall et al. (3); http://www.neurology.org.

The usual measure of the adequacy of the vestibulo-ocular response (VOR) is gain. Gain is a general term to cover the ratio of output to input in any dynamic system. To measure the VOR gain, we calculate the ratio of the area under the eye velocity curve, to the area under the head velocity curve, during the head impulse (4–7) (see Calculating VOR Gain). Normal VOR gain is close to 1.0. Patients with unilateral vestibular loss (UVL) have a reduced VOR gain during the head turn to their affected ear (usually less than 0.7) and so their slow phases do not compensate for head turn, with the result that their eyes move with the head, and they need to make corrective saccades for head impulses toward their affected ear (Figure 4A), thus identifying the side of loss.

Some patients make catch-up saccades during the head impulse, and these are not detectable by the clinician: they are “covert” catch-up saccades (8). They showed the need for an objective record of eye movement and head movement during head impulses. After about 10 years of development, MacDougall et al. came up with a high-speed head-mounted camera on tight-fitting goggles with head velocity sensors, and software for accurate objective measures of the head and eye velocity. The camera measures the center of the pupil, and valid measures require an excellent image of the eye, uncontaminated by the eye lid (see below). This is what has become the widely used video head impulse test (vHIT) (5).

The function of each and every canal can be measured individually with vHIT (4, 9, 10), and there is not much decrement of the VOR with age (11, 12). vHIT can quantify the absolute level of canal function after treatment with systemic (13) or intratympanic gentamicin (14) and measure bilateral loss of vestibular function (15–17) (see Quantitative Head Impulse Test in Central Vestibular Disorders and New Clinical Disease Patterns Emerging from 3D Video Head Impulse Testing). Tracking the pattern of covert saccades after UVL shows that there are changes in the timing and pattern of the saccades which may be related to the adequacy of vestibular compensation. Some patients may use covert saccades to obscure the retinal slip that must occur because of their inadequate VOR (18). vHIT allows for the measurement of the pattern of these covert saccades for addressing the important question of the role of covert saccades in recovery after vestibular loss (19–23).

The vHIT rather than caloric test is becoming the first test for patients with suspected vestibular disorders (24, 25). It is fast, innocuous, repeatable and provides objective quantitative data about each of the SCCs.

Must the loss of SCC function be profound for head impulse testing to detect it? No. The published evidence shows that vHIT may detect mild impairment of canal function. Weber et al. (13) showed that different patients had different levels of bilaterally impaired SCC function on head impulse testing after systemic gentamicin—they did not all show “profound” loss of SCC function, and indeed some patients had small losses in function. The results showed a continuous spectrum of VOR gains on head impulse testing in different patients from almost normal to complete bilateral vestibular loss (BVL). More recently, vHIT has been shown to detect mild loss of function: Marques et al. (14) found a single ITG dose only reduced VOR function using vHIT by an average of 26%—again modestly impaired SCC function rather than being a “profound” loss. Finally, the tight bands of vHIT gain for healthy subjects, which we have published (12), show that even small losses of canal function are detectable. So, just as an audiogram detects a mild impairment of auditory function, so the HIT detects mild impairment of vestibular function.

### The SHIMP Variant

In the standard vHIT, the patient is required to stare at an earth-fixed target during the head impulse. If their VOR is not adequate, the patient must make a corrective saccade to return fixation to the target. vHIT is the measurement system, and we now call the whole protocol HIMP (as abbreviation for Head IMPulse testing). (Since HIMP is a recently introduced term, we will use the terms vHIT and HIMP interchangeably in this review.) In the SHIMP (“Suppression Head IMPulses”) variant, the patient stares at a *head-fixed* target during the head turn (Figure 1B). The head turn stimulus is exactly the same in HIMPs and SHIMPs—brief, passive, unpredictable, high-acceleration head turns—but in the SHIMPs protocol, the subject is required to fixate a target *which moves with the head*—a spot from a head-mounted laser projected on the wall in front of the subject (3). We call this new variant protocol SHIMP because we expected suppression of the VOR to dominate. Healthy subjects suppress their VOR frequently in daily life (reading a book on a bus) but VOR suppression in this passive, high-acceleration vHIT protocol takes around 80 ms from the onset of the head turn (26), so VOR suppression is just starting around the end of the head impulse stimulus. (Note that we have used these new terms since the HIMP and SHIMP protocols can be used with or without the vHIT goggles. SHIMP is even easier to carry out at the bedside than HIMPs, and the corrective saccade is much easier for the clinician to see.)

The results from the SHIMP testing protocol complement the original HIMP protocol: in the SHIMPs protocol it is healthy individuals who make a large corrective saccade (Figure 2B), and the patients without vestibular function who do not make a saccade (Figure 3B). Why do healthy subjects need to make a corrective saccade? Because during the head impulse the VOR drives their eyes opposite to head, and healthy subjects do not suppress their VOR during the early stage (first ~80 ms) of the head turn. Consequently, their gaze is driven by their VOR off the head-fixed target. For example, as the head is turned to the left, the VOR keeps the gaze fixed in space by rotating the eyes in the head to the right, and so at the end of the impulse the target is to the left of the subject’s gaze so the healthy subject has to make a large leftward saccade to regain the target. This is an anticompensatory saccade, since it is in the same direction as head rotation (left). At the other extreme, for a patient with complete vestibular loss (Figure 3B), their absent VOR does not drive their eyes off the head-fixed target at all during the head impulse, so at the end of the impulse the avestibular patient’s eyes are still on target so they do not make any corrective saccade. In SHIMPs, it is the patients without vestibular function who do not make corrective saccades—exactly the converse of HIMPs. Patients with UVL show large SHIMP saccades for head turns to their healthy side and small or absent SHIMP saccades for head turns to their affected side (Figure 4B). In the SHIMPs protocol, the size of the corrective saccade is an indicator of VOR gain—healthy subjects make large saccades, whereas patients with vestibular loss make small saccades or no saccades at all, and a recent study used saccade size as an extra indicator of canal function (27). However, care is needed in interpreting the size of the saccades since there are many factors that affect the peak saccadic eye velocity—for example, the extent of overshoot of head turn, the peak head velocity of the impulse, and even the duration of the head impulse. It is important to avoid any predictive clues about the direction of head turn to avoid an early anticipatory saccade in the direction of head movement. Nevertheless, the results show that saccadic velocity can be used to index SCC function (27). So the SHIMPs protocol provides two measures of SCC function—VOR gain and the size (velocity) of the corrective saccade. VOR gain, measured from the slow-phase eye velocity, is similar for both HIMPs and SHIMPs (3), and SHIMPs has the advantage of effectively removing the covert saccades, which cause problems in gain measurement in the standard HIMPs protocol (see Figure 4).

## The Physiological Basis of the HIT

The basic physiology underlying both HIMPs and SHIMPs is the same. A head turn causes fluid displacement in the SCCs, which deflects the cupula, deflects hair bundles of receptor hair cells, triggers action potentials in primary SCC afferents which project to vestibular nuclei, thence to eye muscle motoneurons of both eyes, and so the resulting conjugate eye movement corrects for the head turn and gaze remains stable even during an unpredictable head turn (28). It is important to understand how the eye movement response is driven and there are some special features:

- The onset of eye movement response is fast—its latency is about 8 ms from onset of the head turn stimulus to the onset of the eye movement response (29).
- The stimulus causes compensatory movement of both eyes, although detailed measures show the two eyes are not exactly conjugate (30).
- The major direct neural pathways governing this response are known.
- Cerebellar input governs transmission through these pathways, and it is that cerebellar input which is responsible for VOR suppression (see also Cerebellum below).

The vHIT provides time series records of the eye movement response to head impulses and of the corrective saccades which occur in patients with UVL. In some patients, there are aspects of the response which initially appear puzzling: for example, why are there compensatory saccades to head impulses to the healthy side in UVL patients (8, 19). This section shows how the established neural connections explain the VOR response in healthy subjects and in patients with UVL. These are not some computer generated fantasies, they are evidence based—built on the hard-won neural evidence.

Rotational testing of SCC function has a major drawback. The one head movement causes complementary stimulation of canals on both sides of the head. The physiology of the canal-ocular pathways shows that for testing patients with low peak head velocities the input from the healthy ear can determine the results for both directions of horizontal rotation. The contribution from the healthy ear can be ruled out by using high-acceleration head impulses, which silence the input from the opposite side. If low velocity (low acceleration) head impulses are used, the remaining healthy ear can drive the eye movement response. That is why high velocity stimuli are mandatory. The essential reason is that for most head impulses the response is made up of two components—a dominant excitatory drive from the side to which the head is turned—and a smaller, indirect, functionally excitatory, drive from the opposite ear.

Normal head movements have very high accelerations—4,000°/s/s and above (31, 32), and so the eye movement response must have a very short latency and be accurate. The response is very fast: about 8 ms from the onset of the head movement stimulus to the onset of the eye movement response (29). It is likely that the onset response is due to the action of the very fast type I receptors and irregular afferents from the central region at the crest of the crista. Receptors of the horizontal canal (HC) are not all uniform—there are two types; type I, amphora shaped receptors with short stiff hair bundles, enveloped by a calyx afferent ending [see Ref. (33, 34) for reviews]. These type I receptors predominate at the crest of the crista and intracellular recordings have shown the very fast dynamic responses of these receptors (35, 36).

**Figure 5:**
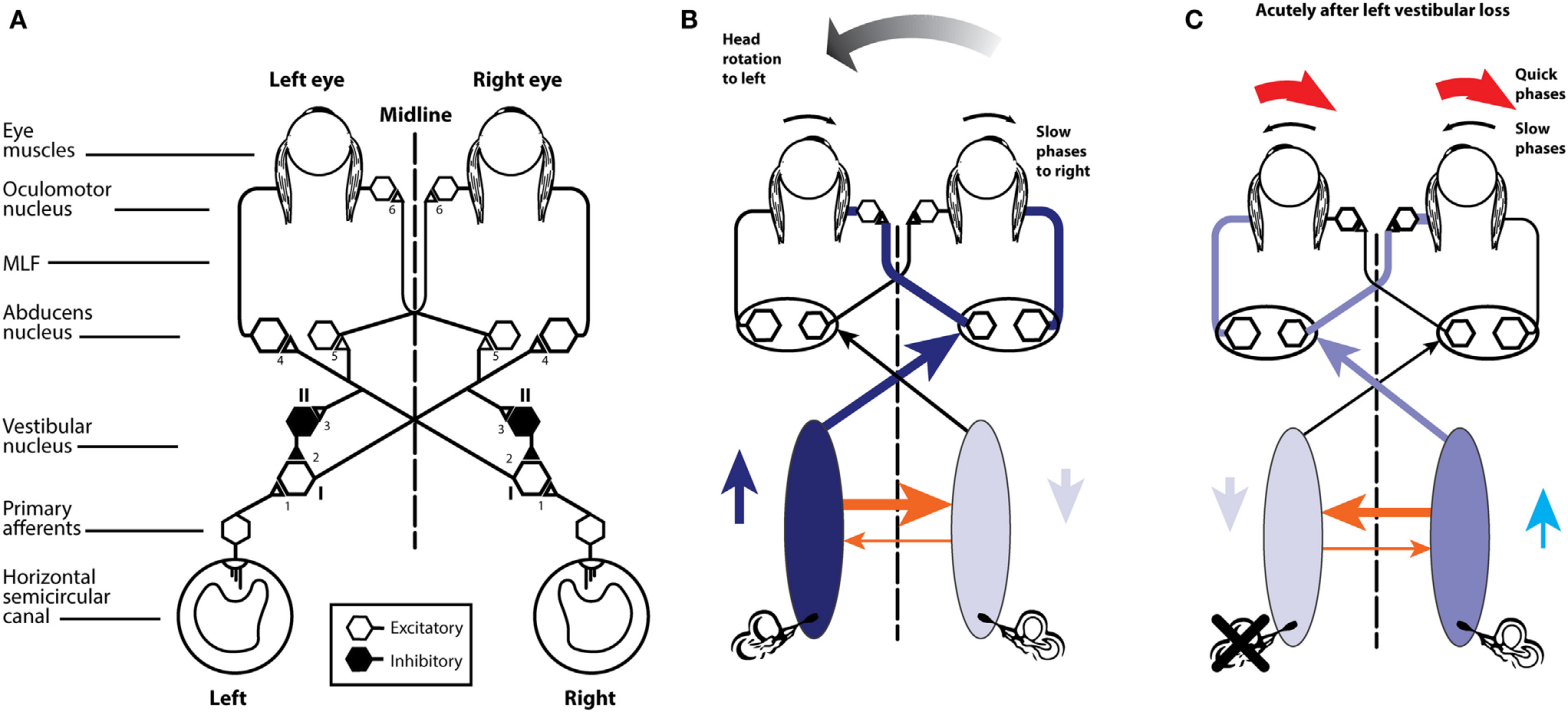
**(A)** Schematic view of the brainstem showing the basic projections underlying the horizontal VOR. The synapses are numbered and the papers giving evidence for each synapse are given in Table 1. Reprinted from Ref. (28), © 1995, with permission from IOS Press. **(B)** A depiction of the neural activity in the VOR network during a head turn to the left, eliciting a compensatory eye movement response to the right (see text for a full description). Increased activation is shown by the darker blue and the thicker orange shows the increased commissural inhibition from the activated vestibular nucleus. **(C)** With head stationary, a unilateral vestibular loss (left here) elicits an imbalance in neural activity between the two vestibular nuclei. The absence of primary vestibular input means that the left vestibular nucleus has reduced activity (light blue), which in turn generates a reduction in commissural inhibition to the right vestibular nucleus (thin orange line), allowing the cells in the right nucleus to fire at a higher firing rate, resulting in the slow phase of vestibular nystagmus to the left and quick phase to the right (red arrows).

The neural pathway for these fast direct projections is shown in Figure 5A. The schema is similar for the following figures that show the consequences of canal activation.

The point of view for these schematic figures is a view looking down on the brainstem and the two eyes. The neural structures involved in the responses are labeled, as are the synapses with each synapse in this direct circuit numbered. The papers providing the evidence about each projection and the synapse are given in the accompanying Table 1. The literature on the anatomy and physiology of the vestibulo-ocular projections is immense, but rather than pointing the reader to a book or a huge review we have listed in Table 1 the selected references for each projection and each synapse.

**Table 1:** Selected references providing the anatomical and physiological evidence for the projections depicted in Figure 5A.

1.	Excitatory projections from HC receptors to type I vestibular nucleus neurons (37–43) (type I neurons increase their firing during ipsilateral horizontal rotations)
2.	Inhibitory projections from type II vestibular nucleus neurons to type I excitatory neurons (44–47) (type II neurons are inhibitory and decrease their firing during ipsilateral horizontal rotations)
3.	Excitatory projections from type I vestibular nucleus neurons to the contralateral vestibular nucleus to inhibitory type II neurons (47–49)
4.	Excitatory projections from type I vestibular nucleus neurons to contralateral abducens motoneurons and internuclear neurons (50–58)
5.	Excitatory projections from type I vestibular neurons to contralateral internuclear neurons (48, 55, 57)
6.	Excitatory projections from abducens internuclear neurons to the contralateral III nucleus (59–62)

Primary afferents from each labyrinth are excitatory and project to the vestibular nuclei; some neurons project from the vestibular nuclei to the contralateral abducens nuclei. One set of axons from abducens project directly to the lateral rectus, another group of abducens neurons (the internuclear neurons) project to the medial rectus of the contralateral eye *via* the medial longitudinal fasciculus (MLF) and the oculomotor nucleus (III). The connections of particular importance for understanding the eye movement to vestibular stimulation are the fibers between the two vestibular nuclei. These are the commissural fibers and they are functionally inhibitory, so each vestibular nucleus acts to inhibit some neurons in the contralateral vestibular nucleus. With head stationary, the activity of the two vestibular nuclei are presumed to be in equilibrium.

There are two other projections of importance which, for simplicity, are not shown here, because their action complements the action of other projections.

(1) Some excitatory type I neurons in the vestibular nucleus project in the ascending tract of Deiter’s (ATD) directly to the ipsilateral III nucleus (63) and so activate the ipsilateral medial rectus (complementing the excitatory input to III from the contralateral abducens internuclear neurons).
(2) Some type I neurons in the vestibular nucleus are inhibitory and project to the ipsilateral abducens nucleus (48) and so complement the decreased input from the contralateral type I vestibular nucleus neurons during head turns.

**Figure 6:**
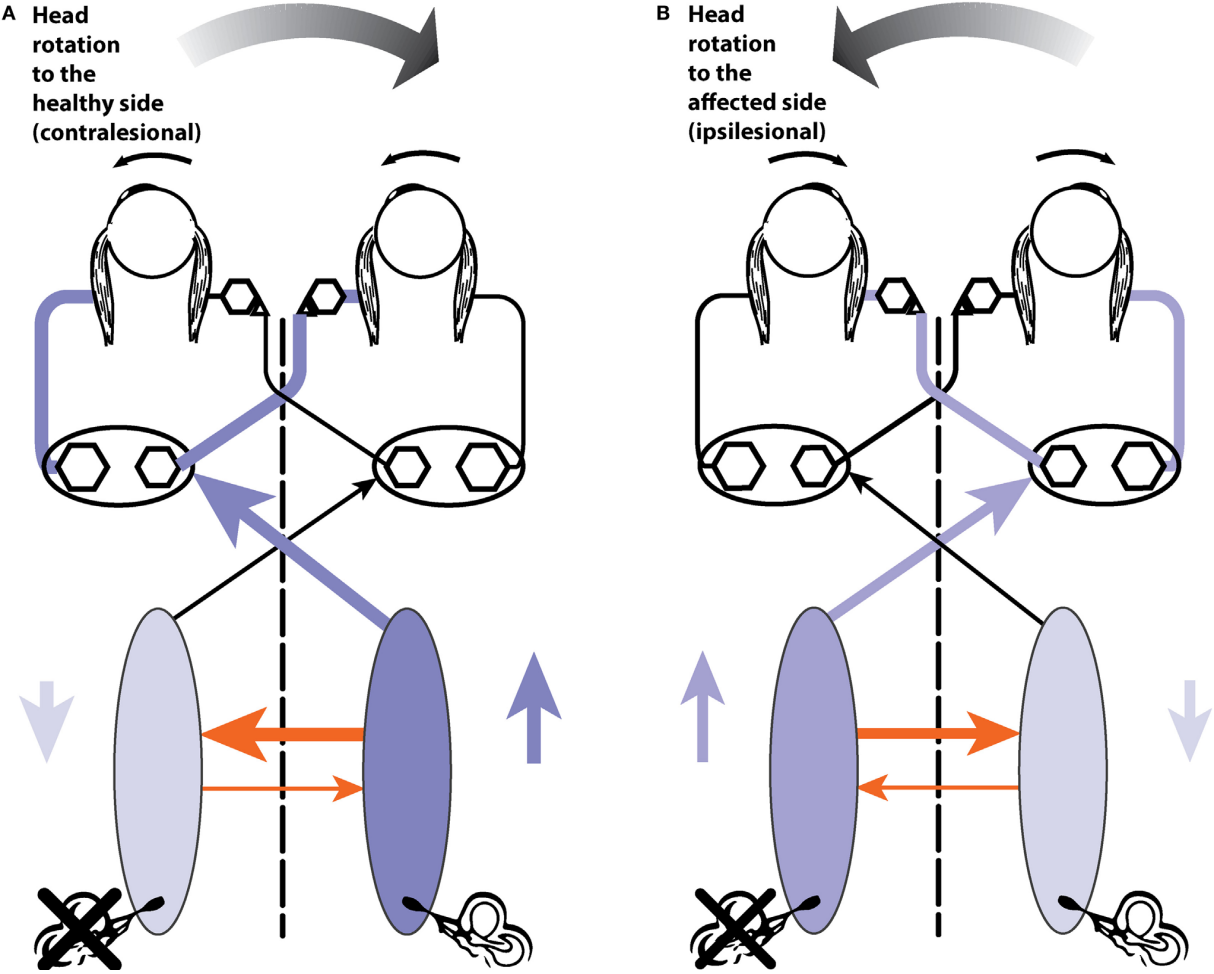
**(A)** A depiction of activity in the pathway during a contralesional (rightward) head turn after a left unilateral vestibular loss. **(B)** Activity during an ipsilesional head turn (see text for a full description).

The aim of the series of images (Figures 5B,C and 6A,B) is to show how the documented anatomical structure and evidence from physiology and neural projection (Figure 5A) proceeds from labyrinth stimulation to eye movement response, how unilateral loss affects the system, and how, after a UVL, the system responds to head turns to the healthy and affected ear. In these figures, the two blue ellipses represent the vestibular nuclei with the darker blue representing increased activation. The orange arrows represent the functionally inhibitory fibers between the two vestibular nuclei and the width of the arrows indicates the strength of the projection.

In a healthy subject during an abrupt head turn to the left, receptor and afferents in the left HC are activated and *simultaneously* receptors and afferents from the right HC are silenced. The excitation of the left labyrinth afferents projects to, and activates, neurons in the left vestibular nucleus, and neurons in that nucleus project activation (blue lines) to the contralateral abducens nucleus, generating the slow-phase eye movement to the right, compensating for head turn. Some neurons in abducens nucleus (so-called internuclear neurons) project *via* the MLF to the contralateral oculomotor nucleus and thus activate the medial rectus muscle of the left eye. In this way, there are synergistic movements of both eyes—both eyes rotate to compensate for head turn and so both eyes remain on the fixation target during the head turn. While these excitatory processes are in action, there are exactly complementary effects exerted by projections from the right labyrinth; the head turn acts to silence the primary afferents from the right SCC, so they project less excitation to the right vestibular nucleus, resulting in reduced excitation of the left abducens and reduced activation of the right medial rectus. So for both eyes, there is simultaneous excitation of one set of eye muscles and simultaneously reduced excitation of the opposing muscles, allowing for a smooth eye movement response of both eyes.

Importantly, however, these direct effects are complemented by the functionally inhibitory connections between the two vestibular nuclei. During the leftward head turn, the excitation of neurons in the left vestibular nucleus acts to further reduce the resting activity of the neurons in the right vestibular nucleus, *via* the functionally inhibitory commissural fibers. But the neurons in the right vestibular nucleus are already firing at a low firing rate because of reduced input from the right SCC. As a result of the reduced activity in the right vestibular nucleus, the neurons in the left vestibular nuclei receive less inhibition from the right vestibular nucleus. In this way, the functionally inhibitory commissural connections act to enhance the difference in neural activity between the two vestibular nuclei.

So the simple excitation from the left labyrinth is complemented by another source of activation—the reduction in inhibition (called disinhibition). As the right horizontal canal decreases activity, cells in the right vestibular nuclei also decrease activity. Thus they exert less inhibition (i.e., they disinhibit) the neurons in the activated left nucleus, allowing these left vestibular nucleus neurons to fire at a higher rate. This is the key—*the normal compensatory eye movement response is due to the combined effect of two functional excitatory components*—*excitation and disinhibition*. Note that the reduced activity in the right vestibular nucleus is enhanced by the increased firing (and thus increased functional inhibition) from the left vestibular nucleus neurons.

If the left labyrinth is damaged (Figure 5C), the afferent neurons from the left labyrinth cease firing and so neurons in the left (ipsilesional) vestibular nucleus have a low firing rate. Thus, these silenced neurons exert less inhibition on cells in the right vestibular nuclei which project to and activate abducens neurons. This reduced inhibition from the left vestibular nucleus allows cells in the right vestibular nucleus to fire at a higher firing rate than their usual resting rate. This imbalance in firing rate between the two vestibular nuclei is equivalent to the imbalance caused by a real head rotation to the right, and the consequence is slow-phase eye movements away from the healthy right side and quick phases away from the affected left side. This is spontaneous vestibular nystagmus with slow phases to the left and quick phases to the right. [The cause of the quick phase is due to a separate group of burst neurons—see Ref. (64).] The higher firing rate of cells in the right vestibular nucleus acts to further inhibit the vestibular nucleus neurons on the left side, which already had their activity decreased by the absence of afferent input from the left labyrinth. Once again the functionally inhibitory commissural neurons act to enhance the imbalance in neural activity between the two vestibular nuclei. Over time the imbalance in neural activity between the two vestibular nuclei reduces and as it does so the spontaneous nystagmus declines as vestibular compensation takes place and equilibrium returns (28).

Now consider testing the horizontal VOR of a patient with a left vestibular loss (Figure 6). First giving them a head turn to their healthy (right) side (6 A). This is also called a contralesional head turn. The stimulus will result in the usual increase in firing of afferents from the remaining right labyrinth projecting to the right vestibular nucleus and thence to the left abducens, to generate a compensatory slow-phase eye movement to the left, just as occurs in response to a rightward head turn in a healthy person, but with one important difference. Now the increase in neural activity in the right vestibular nucleus on the healthy side is not complemented by reduction in inhibition from the lesioned side. The disinhibition is missing. The overall result will be a compensatory eye movement whose eye velocity does not quite match (and so does not exactly compensate for) head velocity, because although it has the excitatory component it is missing the disinhibitory component which healthy subjects have. Because of that absent disinhibitory component the eye position at the end of the head turn to the healthy side will be a little short of the target, so the patient probably will, at the end of this head impulse to the intact side, need to make a (small) compensatory saccade to get their fixation back to target. In other words, there may be a small saccade in UVL patients, even for head turns to their healthy side. This is not a deficiency in the explanation—quite the contrary—it is exactly in line with what is expected by the neural connections that physiological experiments so painstakingly demonstrated. [It is worth noting that this small compensatory saccade will not happen in all patients—it depends on many things—how large is the vestibular loss, how large is the overshoot in the head turn, how big the fixation target is, how well the patient can see the target, and how far off target their final fixation is, and their criterion for deciding if they need to make a saccade at all (is “near enough good enough”).]

The evidence supporting this interpretation based on disinhibition is that for rotations to the healthy side in patients with surgically verified unilateral loss, there is not only a reduction in VOR gain for rotations to the affected side but also a significant reduction in VOR gain for rotations to the healthy side—see Figure 7 of Ref. (65)—often resulting in small overt saccades in UVL patients for rotations to the healthy side (8). The gain reduction is not large but the use of vHIT shows it, because even very small saccades have high velocity and so are easy to detect from the eye velocity records.

Head turns to the lesioned side in a UVL patient will cause an initial compensatory eye movement. Why? When there are no remaining receptors on the left side to be activated. What can cause any compensatory eye movement? The head turn will cause a decrease in activity of receptors in the healthy labyrinth, leading to decreased neural input to the right vestibular nucleus. In turn, this reduction will act to reduce inhibition on cells in the left vestibular nucleus. A reduction in inhibition is an excitatory drive, so the neurons in the left vestibular nuclei will fire at a higher rate and project to abducens neurons and so drive the eye to compensate for the head turn, at least initially. At high head accelerations, this disinhibitory drive will be short lasting—as soon as the neurons in the right vestibular labyrinth and nucleus are driven to silence there can be no further reduction in commissural inhibition, and so the effective excitation will cease. In patients with bilateral loss, there will not be any disinhibition and so there will be no early eye velocity response and the eye velocity records should be flat for both directions of head turn.

It should be noted that there are descending projections from the cerebellum, which can modulate the transmission through the vestibular nucleus and are responsible for the voluntary suppression of the VOR in many situations. However, evidence is that in human subjects in response to abrupt, passive, high-acceleration head impulses, VOR suppression only starts to operate after a delay of around 80 ms (26).

## Practical Aspects and Potential Pitfalls of Video Head Impulse Testing

Video head impulse test allows dynamic testing of vestibular function even in bed-bound patients, without the necessity of a specialized equipment, such as a motorized rotating chair, or the need for special testing conditions—such as total darkness. It is a quick, objective test that can be used to test the dynamic function of all six SCCs (9). It is of special value in testing vestibular function in young children (67–70). The effectiveness of intratympanic injection of gentamicin for treatment of Menière’s disease is now being monitored by vHIT measures of canal function after injection (14). vHIT is now being used to screen potential stroke patients in the emergency room. The vertigo which incoming patients report may be of peripheral origin from VN or central origin from a brainstem or cerebellar stroke. vHIT testing helps resolve this major question: patients with normal VOR on vHIT are more likely to have had a stroke (71).

Many studies have reported the sensitivity and specificity of vHIT with respect to the caloric test. The clear conclusion from such studies is that, not surprisingly, these two different tests give different results. One mistake has been to assume that the caloric is the “gold standard” of horizontal canal function. That is not correct—the caloric is just one test of canal function, just as vHIT is. vHIT uses the natural physiological stimulus of head rotation. The caloric relies on thermal conduction and probably buoyancy to generate a cupula displacement in an artificial, non-physiological manner (because heat is not the natural stimulus for SCC stimulation). The important question is the sensitivity and specificity of vHIT, not against the caloric test, but for detecting surgically verified absent horizontal canal function, and with our colleagues de Waele and Chiarovano, we have shown that the sensitivity and specificity of vHIT for detecting surgically verified absent HC function are both 1.0 (Curthoys, deWaele, and Chiarovano, unpublished data).

It is now clear that many patients (especially those with Menière’s disease) do have normal horizontal canal function—as shown by vHIT, responses to high-acceleration horizontal head rotation, but have reduced or absent caloric responses (72–76). It has been suggested that this dissociation between vHIT and caloric results may be an indicator of Menière’s disease (77, 78). McGarvie et al. (74, 75) have suggested that this dissociation between vHIT and caloric results may occur because hydrops of Menière’s disease dilates the labyrinth and so affects the mechanism by which caloric stimulation activates canal receptors, but that hydrops has little effect on canal-cupula responses to rotation.

In summary, the two testing protocols, HIMPs and SHIMPs, are complementary ways of testing SCC function, and the vHIT system provides the objective records of head velocity and eye velocity for determining the level of semicircular canal function.

### Pitfalls in Video Head Impulse Testing

Since its introduction (4, 5), head impulse testing has become one of the primary frontline vestibular tests in many institutions around the world. The ability to quickly measure the VOR response of all six SCCs in the standard clinical setting has greatly improved our diagnostic accuracy.

However, as with any new test, there are pitfalls for the operators who are not personally experienced with the practical aspects of the test. To minimize these pitfalls, it is important to be aware of what vHIT is measuring since it reduces a complex three-dimensional biological response to one that can be measured with a simpler two-dimensional system. Furthermore, the measurement sensors themselves are mounted in a set of goggles, which are connected to the head in such a way that the measurements truly reflect the head motion in space and the pupil motion within the head. The following outlines some of the potential sources of artifacts and describes techniques to minimize them.

### Head Motion Measurement

A three-dimensional inertial sensor (IMU) is mounted in the goggles, with the primary sensor stimulus planes notionally set along the head stimulus axes [horizontal, left anterior–right posterior (LARP), and right anterior–left posterior (RALP)]. The output of the various systems is the head velocity in a given plane. In order to accurately transduce the head stimulus, two basic requirements need to be met. The first is obviously that the goggles are tightly linked to the skull, as the stimulus is actually applied to the head but is transduced at the goggles level. The second is that the plane of the stimulus matches the appropriate sensing plane of the goggles. For example, if the goggles are mounted on the face such that the horizontal head velocity sensor axis does not match the axis of the applied “horizontal” head impulse, then the measured head stimulus will be lower than the actual head velocity by a factor of the cosine of the angle between the axes. Within a range of ±10° between the axes, this effect is negligible, with the effect being less than 1.5%. However, as the angle increases, the effect becomes more pronounced, reaching 10% at just over 25°. This will artificially increase the gain, calculated as eye velocity divided by head velocity (either instantaneous or over the period of the impulse). Therefore, the stimulus axis and the appropriate sensor axis should be aligned as closely as possible for the most accurate measurement of head motion. It should also be noted that an additional source of variation is the range of orientations of the horizontal canal planes in the skull between individuals—mean and standard deviation of the plane of the horizontal canal with respect to Reid’s baseline is 25.12 ± 5.62° (*n* = 20). So 2SDs give a range of around 11°—ranging from 36 to 14° (79, 80). These sources of variation (81) may contribute to the variation of VOR gain values between individuals.

### Eye Movement Measurement

While actual eye movements are complex three-dimensional rotations with horizontal, vertical, and torsional components, vHIT currently only tracks horizontal and vertical movements of the center of the image of the pupil as captured by the high-speed video cameras. The output of the measurement is eye velocity (horizontally and vertically), as calculated from the motion of the “center of mass” of the pupil image in pixels across the image sensor, combined with the geometrical compensation required when a captured, flat video image represents a three-dimensional eyeball rotation (82). This has various consequences when we are trying to minimize artifacts. The first and most obvious is that the camera and the head motion sensor are both mounted in the goggles. If there is no real movement of head in space or eye in head, but there is a movement of the goggles with respect to the face, it will be measured as both an eye velocity and a head velocity. An example of this is a movement of the goggles pulled by the operator moving the skin on the head during the start of the impulse. During a normal horizontal impulse, this type of effect will produce an artifactual “biphasic” eye velocity superimposed on the actual eye velocity as the goggles “lead,” and then “lag,” the head. The converse may occur if the goggles are way too loose and initially “lag” the skull. It is usually only obvious when there is no or minimal true VOR response (83). Once again, to minimize this, the goggles need to be tightened on the subject’s head as tightly as feasible and the operator should hold the head so that skin movement does not move the goggles on the skull.

**Figure 7:**
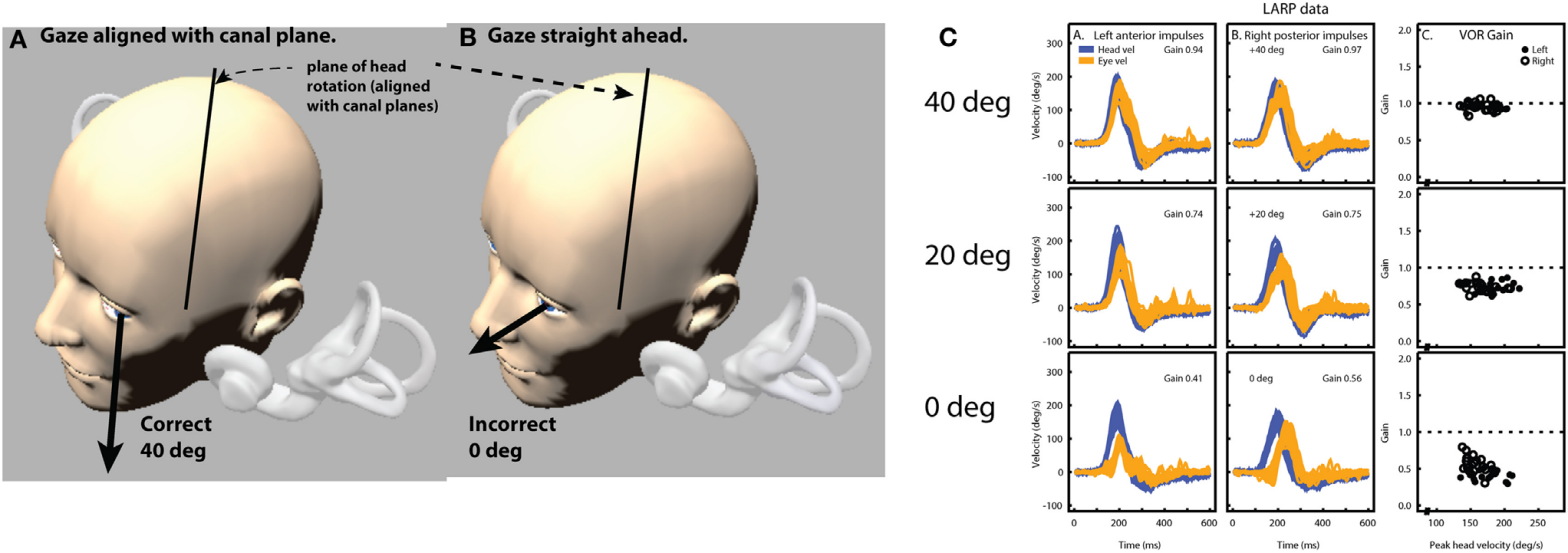
The effect of gaze angle on measurements of the eye movement during vertical head impulses. Because the video camera only measures horizontal and vertical components (not torsional components as yet), it is necessary to arrange the test situation so that that there is minimal contribution of torsion to the eye movement response to vertical canal stimulation (66). This is achieved by gaze being directed along the plane of the canals under test **(A)**. If gaze is straight ahead **(B)**, there is a substantial reduction in vertical eye velocity (and VOR gain) and the reduction is shown in **(C)**—eye velocity from the same subject with only gaze direction changing. Reproduced with permission from the study by McGarvie et al. (10).

For horizontal impulses, the two-dimensional eye velocity measurement does not of itself introduce any significant artifacts. A horizontal head stimulus will elicit a horizontal eye velocity, and gaze elevations or depressions within ±15° will only have a minimal effect on the measured eye velocity (84). However, for the vertical impulses, the situation is more complex, and gaze direction has a major effect on the measured VOR (Figure 7) (10).

For example, a head stimulus in only the LARP plane will produce a vertical response if gaze is also in the LARP plane (66), a roll response if gaze is in the RALP plane and a combined roll and vertical response if gaze is directed straight ahead. Consequently, as the gaze moves away from the plane of stimulation, the measured vertical component of the eye velocity reduces as the roll component increases. There is also a further level of complexity to the vertical impulse response and gaze combination, in that it is physically very difficult to produce a pure LARP stimulus without in any way stimulating the canals of the RALP plane. While the gaze is maintained in the LARP plane, this is not important, as stimulus to the RALP plane will only induce a roll response, which is not measurable. However, as gaze moves out of the LARP plane toward the RALP plane, then a head stimulus that also contains a (small) RALP component will begin to also elicit a vertical eye response, further confounding the measured response. Therefore, it is of major importance to maintain gaze as close to the stimulated head plane as is possible (Figure 7). If gaze does drift out of the plane of stimulus, the results will start to show an apparent “reduced gain” combined with an apparent “delay” of the eye signal, and an absence of corrective vertical saccades.

**Figure 8:**
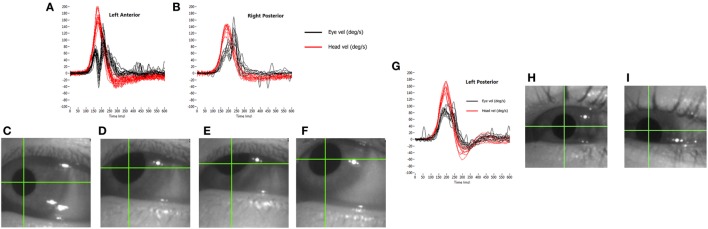
How eyelid obstruction of the pupil during the head impulse generates artifactual eye movement records. **(A,B)** show the results of an eyelid flick touching the top of the pupil: biphasic for the anterior response **(A)** and uniphasic for the posterior response **(B)**. The lower panels show stills from a grossly exaggerated version of how this occurs during the anterior impulse: the pupil starts in the centre of the vertical range **(C)**, then is moving upwards as the eyelid “flicks” down to touch the top of the pupil **(D,E)**, producing an apparent deceleration of the motion. As the eyelid then moves back up **(F)**, the center of mass appears to accelerate upward. **(G–I)** A set of posterior canal impulses **(G)** in the situation where the pupil slides behind a stationary lower eyelid as it moves downward during the stimulus **(H,I)**.

The other major source of artifact, particularly for the vertical tests, is that of eyelid interference with the pupil image. Once again, if we consider the actual output of the test, we can see how the effects occur. With an average impulse at 200°/s, the head motion from takeoff to the zero velocity crossing takes about 150 ms. With camera frame rates of 250–220 frames per second, depending on the system used, about 40 images of the pupil will be collected during the impulse. As eye velocity is calculated from the geometrically compensated change in the position of the “center of mass” of the pupil between successive images, then a small apparent change in this position can lead to a large recorded velocity. The eyelid briefly touching the pupil image can produce a range of artifacts, depending on the stimulus direction and the way in which it interferes with the image (Figure 8).

In our experience, the usual transient eyelid “flick” is the upper eyelid briefly covering the top of the pupil image and then uncovering it, all during the impulse. If an anterior canal (AC) is being stimulated as this occurs, the head is being rotated downward and the eye is rotating upward. As the eyelid touches the top of the pupil, the pupil image will reduce in height and “center of mass” of the pupil image will appear to slow down its upward trajectory. This will reverse as the eyelid uncovers the top of the image, producing an apparent increase in upward velocity. So, an eyelid “flick” during an anterior impulse will produce an apparent, biphasic velocity slow down followed by an apparent speeding up, both superimposed on the actual upward eye velocity. If this eyelid “flick” happens during a posterior impulse (which is a rarer situation), then the eye is moving downward during the impulse. As the upper eyelid touches the top of the pupil image, the “center of mass” will appear to move even further downward, with an apparent rapid increase in velocity, returning to the true eye velocity as the pupil is uncovered. This can have the appearance of a covert saccade superimposed on the true vertical eye velocity, as in this case the effect is uniphasic as pupil and upper eyelid are initially moving in the same direction. Both of these situations can be seen in Figure 8, an extreme case of a subject with a normal VOR and a reflexive large, quick “flick down” of the upper eyelid in response to each impulse.

Although these artifacts appear obvious on the velocity traces, they are almost impossible to see by eye on the video image as they happen so quickly. More recent systems allow recording and replay of the video image, which should be used if these artifacts are suspected.

A variation of these effects occurs when the eyelid does not “flick,” but the pupil slides under it as the eye moves upward during an anterior stimulus. In this case, the eye velocity curve can look like a “table-top,” with a velocity plateau during the impulse. This is due to the “center of mass” still appearing to move upward as the pupil image reduces in height until the head velocity drops to a point at which the two signals rematch. A clue to the artifactual nature of this type of eyelid effect is a “table-top” like eye velocity profile, with no catch-up saccades after the impulse. The same situation can occur with the pupil running behind the lower eyelid as the eye moves downward during a posterior head impulse, as shown in Figure 8. Once again, use the playback of the video recording to confirm these artifacts, and aggressively tuck the eyelids out of the way to avoid such situations.

### Basic Geometrical Considerations

The final factors to consider when optimizing the vHIT are the basic geometrical considerations of the test. Even though head rotation is the primary stimulus, the head rotation axis rarely occurs around the eye actually being measured. As such, in addition to the rotation response, there is a variable, within-space translation of the eye combined with the gain-changing effect of target distance. In comparison between subjects, these effects can be noticeable. The geometrical difference in the stimulus between a subject with an upright flexible neck and a subject with a head carried forward and a stiff neck can be appreciable, particularly when we consider that the eye being measured is always to one side of the center line to the target. While these effects are usually small, an operator wishing to optimize the test output should bear them in mind and adjust the test environment accordingly.

Taking note of the basics of how the test works and the factors that are actually measured will hopefully improve the diagnostic accuracy and ease of the video head impulse in the years ahead. The ability to measure the VOR response of all six SCCs is a dramatic step forward in enabling us to help our dizzy patients.

## Calculating VOR Gain

While the vHIT has become a valuable addition to vestibular testing—one issue remains controversial: the method for calculating VOR gain (7). For vHIT, we calculate the gain of the slow-phase VOR response by comparing head and eye velocity using a “wide window” from the beginning of the impulse until the head velocity returns to (or crosses) 0°/s (4). Instantaneous or “narrow-window” gain calculations that were traditionally used for search-coil recordings are not reliable for vHIT because some goggle movement is practically unavoidable when manually delivering passive head impulses using force applied *via* the flexible skin and flesh. The “wide window” method is less sensitive to movement of the goggles with respect to the skull (and eyes) because these movements are biphasic, with an acceleration and deceleration component that tend to cancel out.

**Figure 9:**
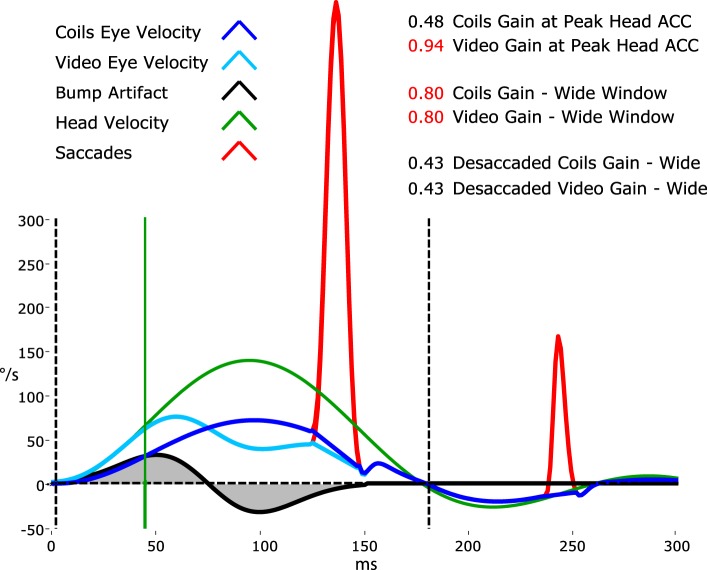
A model of head impulse gain calculation. The figure shows a slow-phase eye velocity response recorded by search coils (blue trace) and by video—video head impulse test (vHIT) (cyan trace) with compensatory “catch-up” saccades (red). The difference between these two traces is the goggle movement “bump artifact” (black trace) that is practically unavoidable for any video goggle system. Traditional VOR gain measurements over a narrow window that is usually centered on peak head acceleration (vertical green line) are very sensitive to contamination by this artifact (red gain values). For vHIT, we measure gain over a “wide window” from the beginning of the head impulse until the head velocity returns to 0°/s (vertical black dashed lines). This gain calculation method is relatively unaffected by the biphasic “bump artifact” (gray shaded areas) because the positive component (caused by manual acceleration of the head) and the negative component (deceleration) tend to cancel out during the impulse. Gains calculated using this wide window method are very similar for video and coils and quite comparable to the traditional narrow window gain measurement method for search coils.

Figure 9 shows an exaggerated model of the “Bump Artifact” (gray), which affects any video goggle system. In this example, a calculation of gain using the traditional search-coil method (with a narrow window at peak head acceleration—vertical green line) produces large errors (nominal values shown in red). Similarly, an instantaneous or narrow-window gain calculation around any other point (e.g. peak head velocity) will also produce errors. The exception to this would be the point at ~75 ms where, in this example, the coil eye velocity (blue) and video eye velocity (cyan) cross over (have the same value). Unfortunately, it is not possible to know the latency of this point (which varies between trials, subjects, and operators) without simultaneous search-coil recordings, which are impractical for routine clinical applications.

Simultaneous search-coil and video recordings (on the same eye) are feasible in the laboratory and provide: a powerful tool for validating vHIT systems; an objective measure of the “Bump Artifact”; and a method to compare the performance of various gain calculation methods (5). Without simultaneous search-coil recordings, a vHIT system developer or user might easily remain ignorant to the existence of the problem and effectiveness of the proposed solution. In 2014, Agrawal et al. (85) attempted to validate an instantaneous gain calculation using simultaneous search-coil measures. Unfortunately, they measured the right eye with a search coil and the left eye with video rather than doing simultaneous recordings of the same eye. Since we know that the eye-movement responses during head impulses are not completely conjugate (30), this instantaneous gain validation was rather less compelling.

A simple position gain is calculated from the ratio of the areas under the desaccaded eye velocity and head velocity traces after “covert” saccades are removed. This simplified rotational position gain is a convenient and sufficiently accurate approximation of the actual eye rotation required to maintain fixation on a target at least 1 m in front of the subject for horizontal and vertical head impulses (9). In other situations, for example with a close fixation point, this simplification can be less than adequate. A more sophisticated conception of gain that can be used with many different head movements and target positions would be one that compares measured eye movement responses and the ideal eye movement response that would be required to maintain fixation.

**Figure 10:**
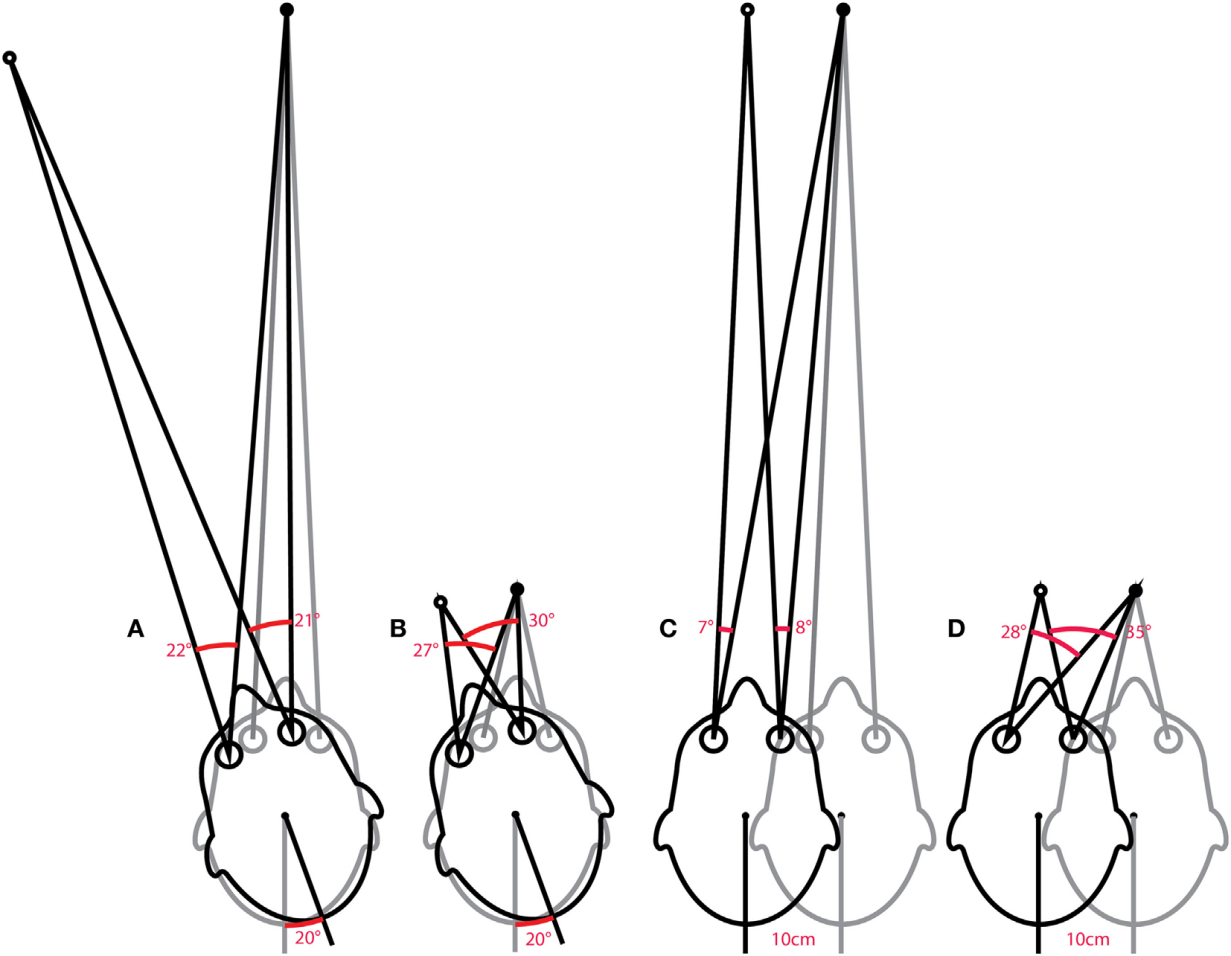
Issues in how VOR performance should be calculated. The ideal eye movement response is one that is compensatory (i.e., maintains fixation) during head impulses with a fixation point: far **(A)** and close **(B)**, and for linear “head heaves”: far **(C)** and close **(D)**. Even in this simple example in one (horizontal) plane, with head rotation around a single point (or for purely linear translations), the calculation of an ideal eye movement response must factor in the geometric consequences of fixation distance, gaze eccentricity, interocular distance, head size (rotation radius), etc. The ideal eye movement rotation responses for a 20° head rotation and a 10 cm head translation are different: for the head rotation for the two eyes, and for the two fixation distances. With more natural head movements, in six degrees-of-freedom, these calculations are more complicated. Although technologies to track head movements and target positions in six degrees-of-freedom are improving rapidly, it is still convenient for video head impulse test to approximate gain calculations with simplified head rotation vs eye-rotation calculations. Such a convenient simplification does however require an understanding of the limitations and some diligence in minimizing departures from the assumptions such as a distant fixation point.

An “ideal” gain calculation factors in the geometric consequences of fixation distance but also of eye translation, which is part of any head rotation around an axis that is usually offset from one or both eyes. With the single exception of head pitch around an axis that passes through the center of both eyes, all head rotations produce some translation of the eyes. Figure 10A shows a very simple example of horizontal head rotation (around the *Z* axis). With a fixation point closer than 1 m (the minimum suggested distance), a 20° rotation of the head (around its center) would require an ideal eye movement rotation response of ~22° in the left eye and ~21° in the right eye. The difference between the head and eye rotations is caused by the translation of the eyes during the head rotation. With a fixation point further than a meter, these differences become less significant and the simplified calculation of gain using eye rotation vs head rotation is acceptable. In other situations such as the very close fixation point shown in Figure 10B, a simple gain calculation would not be sufficient and gain would best be calculated as ratio of measured slow-phase eye response to the ideal eye responses of ~27° and ~30° in the left and right eyes, respectively.

The issues concerning the calculation of gain vs the ideal eye movement response is particularly clear when considering head “heaves” or linear head movements (86), where a comparison between eye rotation and head rotation would obviously be meaningless. Figures 10C,D show linear head movements with far and close fixation points and very different “ideal” eye movement rotation responses required to maintain fixation. With these linear head movements, it only really makes sense to calculate gain as a ratio of measured eye movement response to the ideal eye movement response, and since almost every head movement (including “pure” rotations) involves some translation of the eyes it would be beneficial to consider these geometric consequences in various situations. Considering the geometry and ideal gain would help in the interpretation of small differences in simple gain between the two eyes, between different head turn directions, for different head turn axes (LARP, RALP), with unusual fixation distances and eccentricities, and where gain and catch-up saccade patterns do not match perfectly. Ideal gain calculations require accurate measurement of (or valid assumptions about) the geometry of head movements and target locations in six degrees-of-freedom, so in practice it is often better to keep simple gain calculations valid by understanding the limitations of the idealizations on which they are based, and not to interpret departures from these assumptions as indicators of vestibular dysfunction.

## Quantitative HIT in Central Vestibular Disorders

Central vestibular disorders are caused by lesions in the vestibular pathways, which include vestibular nuclei in the brainstem, ascending projections in the MLF, vestibulocerebellum, thalamus, and parieto-insular vestibular cortex (87). Clinical manifestations include vertigo, imbalance, and localizing neurological deficits. Lesions vary in nature but may be due to acute ischemia, demyelination, and metabolic disorders.

Clinical HIT can be performed quickly for individual SCCs to qualitatively assess the vestibulo-ocular reflex (2). It is the most important test in differentiating central from peripheral vestibular disorders, especially when traditional neurological deficits may be subtle or absent, such as in acute vestibular syndrome (AVS) (88, 89), subclinical internuclear ophthalmoplegia (90, 91), and early Wernicke’s encephalopathy (92, 93). HIT gain and compensatory saccades can be quantified using search coils (8, 94), or high-speed video-oculography (VOG) (4, 5). In this review, we discuss some advances in quantitative HIT in central vestibular disorders.

Published research articles employ either search coil and/or VOG to record HIT in central vestibular disorders. For search-coil studies, there are binocular or monocular (left eye) recordings, and horizontal-only or individual SCC plane HIT (95). For VOG studies, there are monocular (right eye) recording and horizontal-only or modified-individual SCC plane HIT (66). Search coil is the gold standard in eye movement recording, but is semi-invasive and non-portable. VOG has inferior spatiotemporal resolution but is non-invasive and portable.

### Acute Vestibular Syndrome

**Figure 11:**
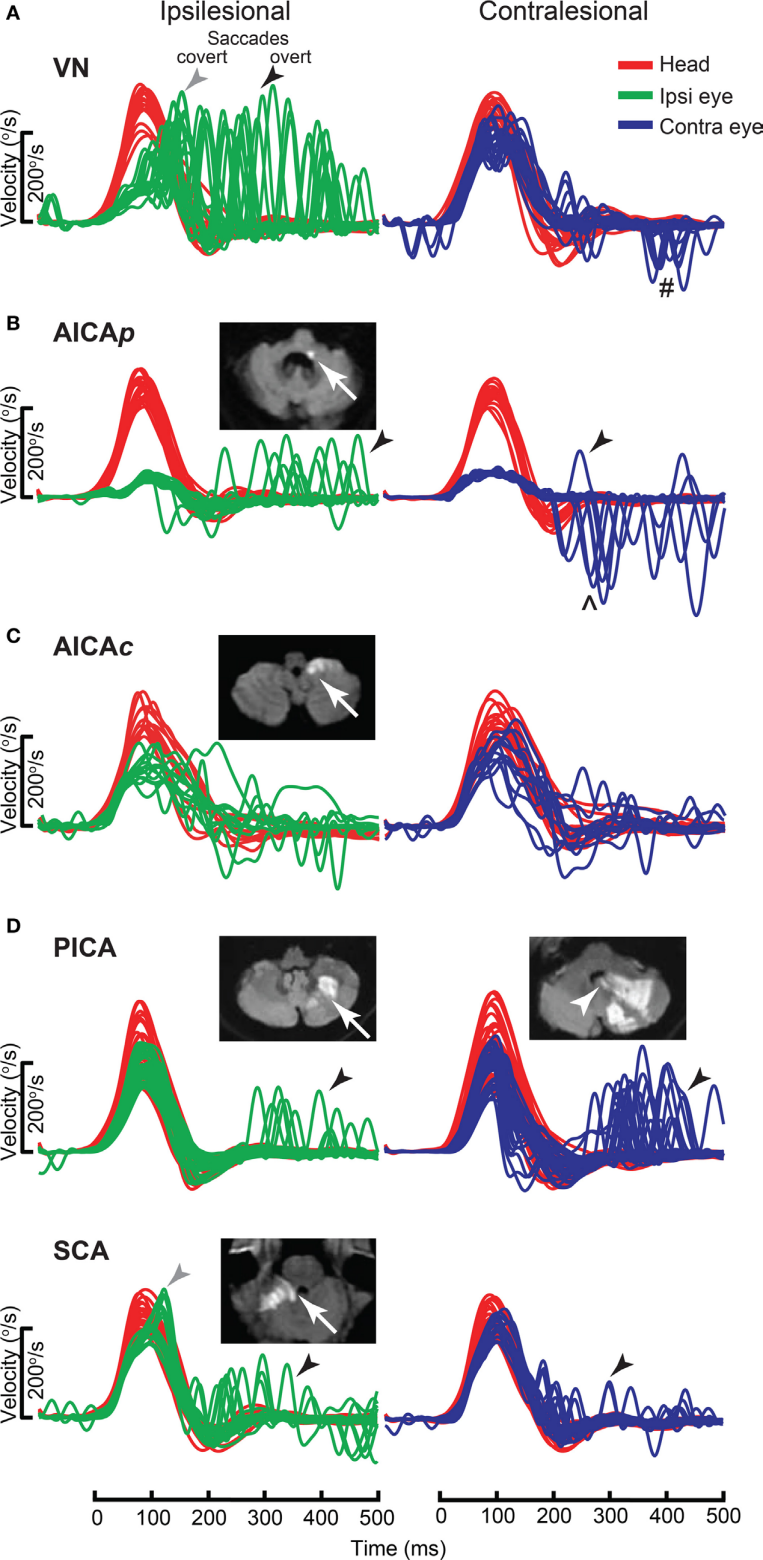
Examples of head impulse test (HIT) results. (1) Posterior circulation stroke (PCS) and vestibular neuritis (VN). Examples of HIT in PCS and VN, displayed as time series of inverted eye (ipsilesional: green, contralesional: blue) to head (red) impulse velocities. **(A)** In VN, ipsilateral gain deficit (mean 0.16) led to large overt (black arrows) saccades (cumulative amplitude: 9.1°, mean) and frequent covert saccades (73% of trials). Contralesional gain was mildly reduced (0.72), matched by small overt saccades (1.2°). Saccades occurring in the direction of contralesional impulses (#) were quick phases of spontaneous nystagmus. **(B)** In anterior inferior cerebellar artery-peripheral (AICA*p*) stroke due to left vestibular nuclear infarction (white arrow), gains were bilaterally deficient (ipsilesional: 0.11, contralesional: 0.21) and overt saccades were present bilaterally, larger after ipsilesional (5.7°) than contralesional (3.3°) trials. Compared to VN, overt saccades were 63% smaller after ipsilesional trials but 2.8 times larger after contralesional trials. Anticompensatory saccades (^∧^) were dominant after contralesional trials. **(C)** In anterior inferior cerebellar artery-central (AICA*c*) stroke due to isolated right floccular infarction, gains were asymmetrically reduced (ipsilesional: 0.55, contralesional: 0.75) with few small overt saccades (ipsilesional trials: 2.7°, contralesional trials: 2.1°). **(D)** Upper: in posterior inferior cerebellar artery (PICA) stroke involving the left cerebellar hemisphere and nodulus (white arrowhead), gains were symmetrical (ipsilesional: 0.85, contralesional: 0.82) with frequent overt saccades, larger after contralesional (4.3°) than ipsilesional (2.8°) trials. Lower: in superior cerebellar artery (SCA) stroke involving the superior vermis, gains were mildly reduced bilaterally (ipsilesional: 0.66, contralesional: 0.71) with small overt saccades (ipsilesional trials: 2.2°, contralesional trials: 1.2°). Reproduced from the study by Chen et al. (104), used with permission from Wolters Kluwer Health, Inc.

Acute vestibular syndrome is commonly due to VN (96, 97) but is closely mimicked by posterior circulation stroke (PCS) (98, 99). A negative clinical horizontal HIT, or the absence of compensatory saccade, is a strong predictor of PCS (88, 89) but its interpretation is dependent on examiner experience (100). vHIT gain can differentiate VN from PCS with sensitivity of 88% and specificity of 92% (101), which is comparable to clinical HIT (102, 103). Search-coil measurement yields better sensitivity of 94–97% and specificity of 90–100% for detecting PCS (104). It demonstrates a spectrum of horizontal VOR gain and compensatory saccade abnormalities in different subgroups of PCS compared to VN. In contrast to unilateral gain deficit and corresponding large compensatory saccade amplitude, PCS involving the anterior inferior cerebellar artery (AICA) territory (AICA stroke) leads to more symmetric bilateral VOR gain reduction and smaller saccades, while posterior inferior cerebellar artery (PICA) territory PCS (PICA stroke) typically leads to symmetrical mild reduction in VOR gain with smallest saccades (Figure 11).

Anterior inferior cerebellar artery stroke causes ipsilateral vestibular loss, conceivably due to labyrinth and lateral pontine involvement (105, 106); the unexpected contralateral gain reduction is speculated to involve the inhibitory and excitatory projections of the floccular target neurons in the ipsilateral vestibular nuclei, reciprocal commissural inhibitory inter-vestibular nuclei connections, and possibly adaptive changes in the flocculus (107). PICA stroke, which sometimes affects nodulus/uvulus, causes on average 20% reduction in gain, a finding that has been demonstrated with search coil (104) but not VOG (108). Such reduction is only modest, perhaps because only 20% of nodulus targeting neurons, including position-velocity-pause neurons, in the vestibular nuclei, are sensitive to eye movement (109).

In VN, saccade amplitude (mean: ipsilateral 8.5°, contralateral 1.3°) and asymmetry are expected to mirror gain reduction (mean: ipsilateral 0.22, contralateral 0.76) and asymmetry. In AICA stroke, although ipsilesional gain reduction is similar to VN, saccade amplitude is smaller, perhaps invoking the flocculus that can influence saccades (110, 111). Experimental lesion of the flocculus causes postsaccadic drift, which can be backward (112). As contralateral gain is reduced more than occurs in VN, saccade amplitude is correspondingly larger. Thus, AICA stroke could produce bilaterally positive clinical HIT (mean: ipsilateral 4.7°, contralateral 3.3°), since clinical detection threshold varies from 1–2° (113) to 3–4° (104). The practical implication is that bilateral positive clinical HIT does not localize to only the peripheral vestibular system (108), and AICA stroke should be considered in the differential diagnosis. Finally in PICA stroke, saccades occur more frequently and/or are collectively larger during contralateral HIT, potentially representing refixating eye movements as a result of dorsal vermal lesion causing ipsilesional saccadic hypometria (114, 115).

### Specific Central Vestibular Lesions

#### Cerebellum

Cerebellar lesions have variable effect on VOR. Diffuse or degenerative processes can cause asymmetric VOR gain, higher for anterior SCC (AC), and can alter the axis of eye rotation (116). Whereas horizontal VOR evoked by rotation is increased in both ataxia telangiectasia (117) and spinocerebellar ataxia type 6 (SCA 6), it is decreased in response to horizontal (118) and individual SCC HIT in SCA 6 (119). Such selectivity for stimulus frequency is perhaps related to degeneration of flocculus (107), and vestibular nucleus, where processing of linear and non-linear pathway inputs (120, 121) might take place. Finally, acute unilateral tonsillar lesion does not affect VOR gain (122). Anatomical–physiological correlation in focal cerebellar lesions may not be exact, since non-lesioned parts of cerebellum may participate in learning and adaptation.

#### Nucleus Prepositus Hypoglossi (NPH)

Acute lesion of NPH produces a distinctive pattern of abnormal SCC function, resulting in contralateral horizontal SCC (HC) hypofunction but bilateral AC hyperfunction (123). Contralateral HC impairment may be explained by a loop of stronger crossed inhibitory projections from NPH to inferior olivary nucleus (ION), inter-NPH inhibitory connections and inhibitory climbing fibers from ION reaching contralateral flocculus. Based on these known connections, ipsilateral NPH lesion could result in increased inhibition of ipsilesional ION, disinhibition of contralateral flocculus, and increased inhibition of contralesional vestibular nucleus. The bilateral AC hyperfunction can also be explained by the same loop, since ipsilateral AC pathway is preferentially inhibited by flocculus (124).

#### Vestibular Nucleus

Acute vestibular nucleus lesion leads to UVL, selective for HC and posterior canal (PC) while sparing the AC (125). Since the anterior SCC afferents project to both superior as well as medial vestibular nuclei (126, 127), lesion of medial vestibular nuclei could possibly only affect HC and PC function. Contralesional HC and PC function is also reduced, possibly due to adaptive change mediated by inhibitory interneurons.

#### Medial Longitudinal Fasciculus

Disruption of the MLF produces internuclear ophthalmplegia (INO), a dysconjugate horizontal eye movement disorder classically characterized by abducting eye nystagmus and adducting eye slowing during horizontal saccades (128–130). Such dysconjugacy can be clinically subtle or silent, but can be quantified by a versional dysconjugacy index (91, 131). The horizontal SCC afferents enter the medial vestibular nuclei, which send projections to the contralateral abducens nucleus, from which two projections arise: directly to the contralateral lateral rectus, responsible for abduction, and *via* crossed abducens interneurons ascending in ipsilateral MLF, responsible for adduction (Figure 5A). There is an extra-MLF pathway for horizontal VOR that arises from the lateral vestibular nucleus and projects to the ipsilateral oculomotor nucleus *via* ATD (63, 132, 133). The MLF also carries projections from anterior and posterior SCCs, but some secondary neurons in superior vestibular nucleus that received projection from anterior SCC travel outside the MLF in the crossed central tegmental tract or brachium conjunctivum (134). The functional consequence is that contralateral posterior SCC function is selectively impaired relative to anterior SCC function, as demonstrated in a case of acute unilateral INO (135).

**Figure 12:**
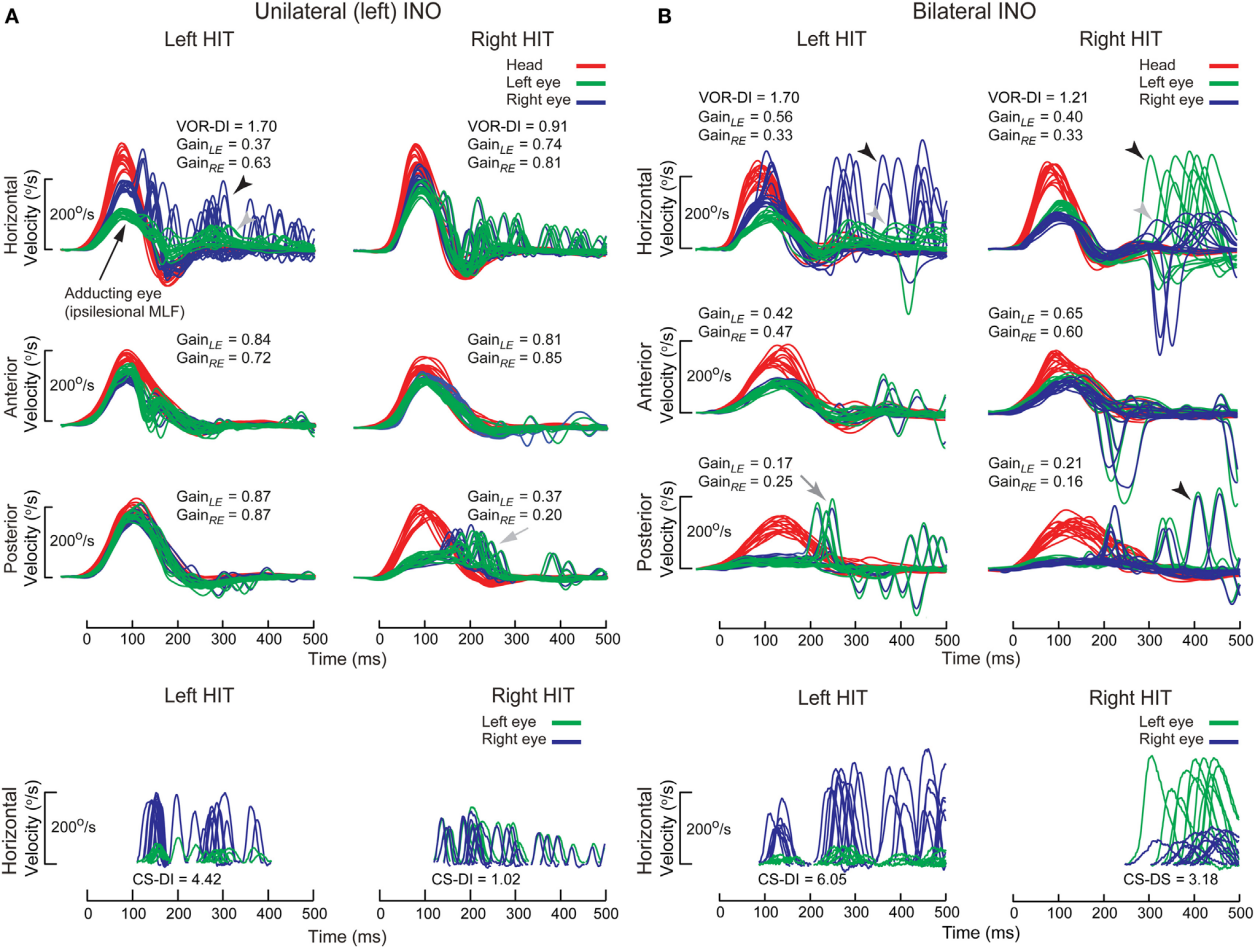
Examples of head impulse test (HIT) results. (2) Internuclear ophthalmoplegia. Binocular search-coil recording of head (red) and eye (left: green, right: blue) in internuclear ophthalmoplegia. **(A)** Top: in left internuclear ophthalmplegia (INO) during ipsilesional horizontal canal (HC) (i.e., leftward) plane (HC) impulses, vestibulo-ocular reflex (VOR) was dysconjugate: gains were lower for the adducting than abducting eye, as measured by the VOR dysconjugacy index (VOR-DI), the ratio of abducting to adducting eye gain. During contralesional HC impulses, conjugacy was maintained. Abducting eye gains during either HC impulses were mildly reduced, possibly due to additional partial abducens nerve or supranuclear gaze involvement. All vertical canal function was preserved except for contralesional posterior canal (PC). Bottom: saccades elicited during ipsilesional HC impulses were also dysconjugate, as measured by the compensatory saccade dysconjugacy index (CS-DI), but more severely affected than VOR-DI. **(B)** Top: in bilateral INO during HC impulses to either side, dysconjugacy was present, albeit asymmetrically in this case. Abducting eye gains were lower compared to unilateral INO, possibly due to defective disfacilitation of the medial rectus motoneurons by the excitatory abducens interneurons, which are normally inhibited by type 1 vestibular neurons. All vertical canal function was impaired, but anterior canal was relatively less affected than PC. Bottom: like in unilateral INO, CS-DI was more severely affected than VOR-DI.

In both unilateral and bilateral INOs (Figure 12), horizontal VOR dysconjugacy is less severe than compensatory saccade dysconjugacy. This discrepancy might be because of some contribution of ATD in driving adduction during VOR, relative to no contribution from the damaged MLF in driving adduction during saccade. Vertical–torsional VOR is impaired, both for anterior and posterior SCC, but is relatively more severe for posterior SCC. This dissociation between anterior and posterior SCC function is less obvious in bilateral INO, potentially related to relative strength of extra-MLF pathways for anterior SCC signals (136), on–off direction asymmetry of the paired vertical SCC (137) and higher sensitivity of secondary neurons to angular acceleration (138).

### Metabolic Disorders

#### Acute Wernicke’s Encephalopathy (aWE)

**Figure 13:**
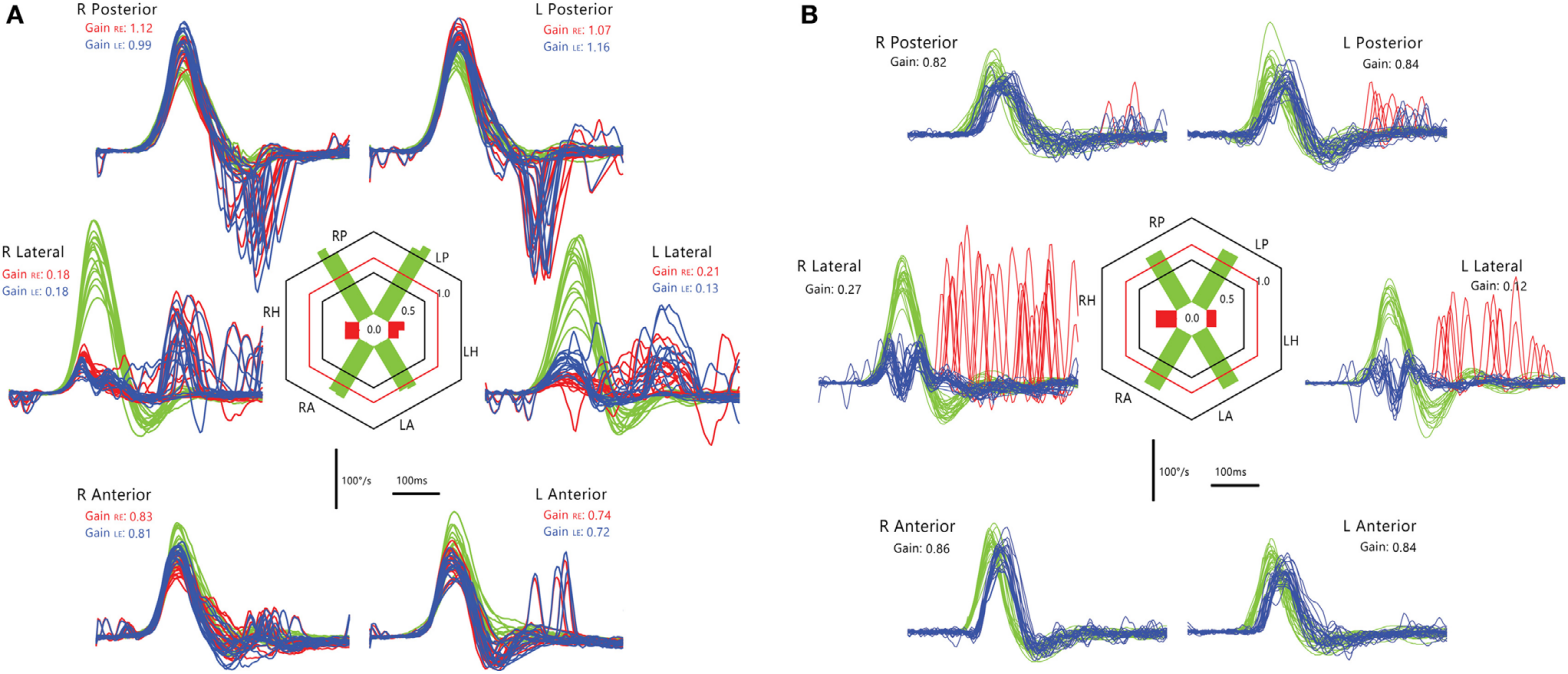
Examples of head impulse test (HIT) results. (3) Acute Wernicke’s encephalopathy. **(A)** Binocular search-coil recording of head (green) and eye (left: red, right: blue) velocity demonstrating severe loss of horizontal canal (HC) function with slow, overt saccades, but preservation of vertical canal function during individual canal plane HIT. Increased posterior canal function with anticompensatory saccades might be related to the presence of spontaneous upbeat nystagmus. Hexagonal bars depict gains from each canal. **(B)** Monocular, right eye (blue) video HIT recording of another patient, again demonstrating severe loss of HC function with preservation of vertical canal function. Reproduced from the study by Akdal et al. (93), used with permission from Elsevier.

Acute Wernicke’s encephalopathy, a medical emergency, is due to thiamine deficiency, and if untreated leads to permanent neurological deficits (139). The classic triad of altered mental status, ocular motor abnormality, and ataxia may not be present, and form fruste cases without overt encephalopathy present with acute bilateral vestibular loss (BVL) selectively affecting horizontal but not vertical VOR (92). Such dissociation might be characteristic of aWE, as has been demonstrated by search-coil and VOG recordings of individual SCC plane HIT (93, 140) (Figure 13). Presumably, thiamine deficiency has selective and profound detrimental effect on the medial vestibular nucleus. Rapid recognition and prompt treatment with thiamine replacement can improve neurological function.

#### Gaucher’s Disease (GD)

**Figure 14:**
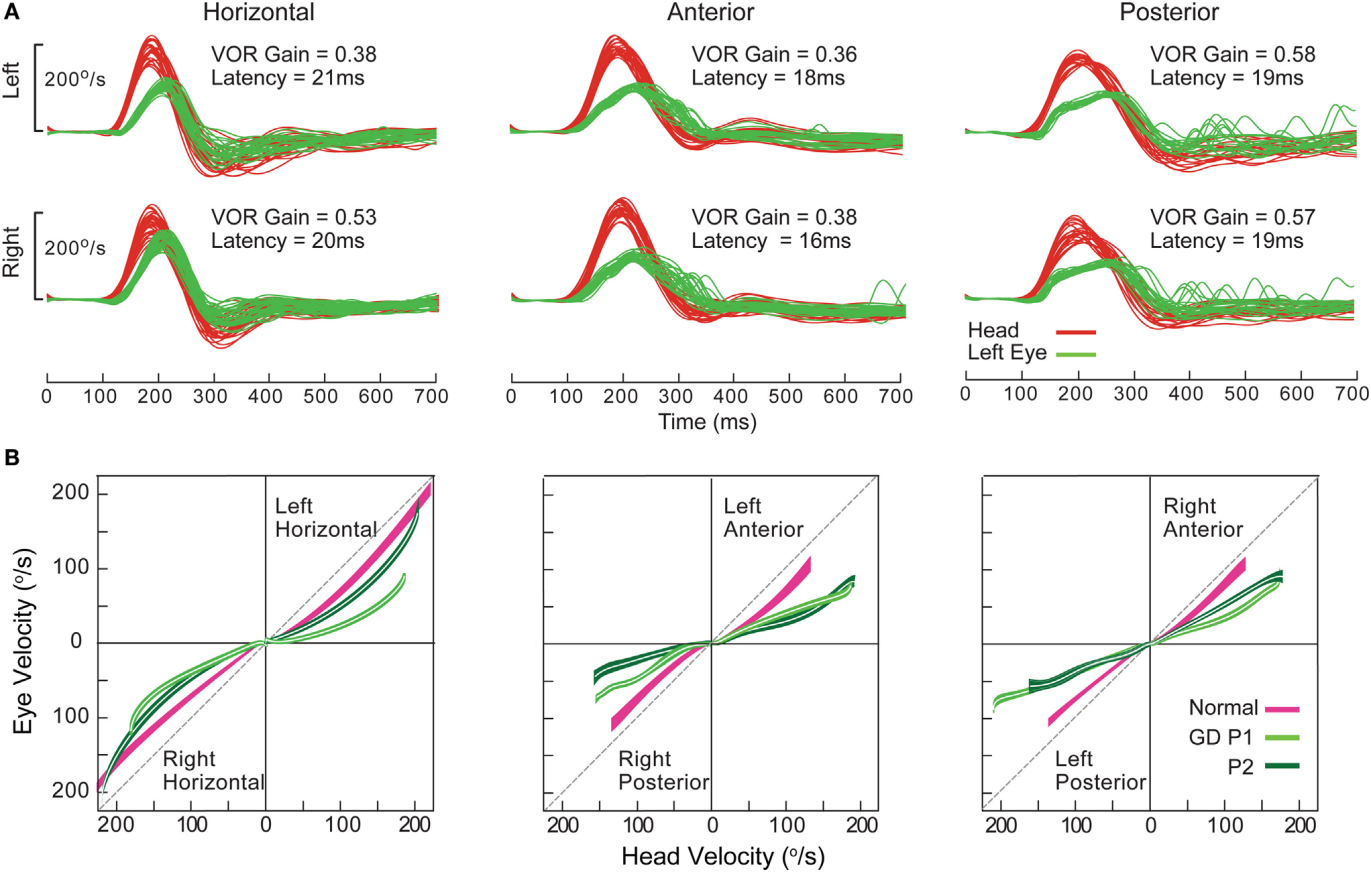
Examples of head impulse test results. (4) Gaucher’s disease (GD). **(A)** Monocular search-coil recording of head (red) and left eye in patient 1 (P1) with GD. Gains for each canal were substantially reduced, but there was a paucity of compensatory saccades especially during horizontal canal impulses. Vestibulo-ocular reflex (VOR) onset latency was prolonged. **(B)** Phase-plane plots of eye versus head velocity for normal (pink), patient 1 (P1, light green), and patient 2 (P2, dark green). Normal phase-plane plot is slightly curved but approximates the diagonal (dotted gray line), indicating near matching of eye to head velocity. In both P1 and P2, due to gain deficit and latency delay, the curve is markedly deviated from the diagonal. Reproduced from the study by Chen et al. (143), used with permission from Springer.

Gaucher’s disease is a hereditary metabolic disorder due to glucocerebrosidase deficiency leading to multi-organ deposition of glucocerebroside (141). Its hallmark eye movement abnormality is saccade slowing, more selectively for horizontal than vertical saccades (142, 143). HIT demonstrates both horizontal and vertical VOR loss and prolonged VOR onset latency, with very small compensatory saccades during horizontal HIT and relatively more frequent saccades during vertical HIT (Figure 14). The BVL is likely due to neuronal loss in the vestibular nuclei (141), while preferential involvement of horizontal over vertical saccades could be ascribed to relatively more severe involvement of pontine paramedian reticular formation (horizontal saccade generator) over rostral interstitial of the MLF (vertical saccade generator). However, this is not the case in postmortem examination, presumably because of the advanced disease stage (144).

## New Clinical Disease Patterns Emerging from 3D Video Head Impulse Testing

With the widespread use of vHIT for all six SCCs in daily clinical practice, not only are classic vestibular disease patterns, such as superior vestibular neuritis (VN) confirmed, but also new and exciting disease patterns are emerging. Together with cervical vestibular-evoked myogenic potentials (cVEMPs) for measuring saccular function, ocular vestibular evoked myogenic potentials (oVEMPs) for measuring utricular function and audiometry for testing cochlear input, it is now possible to assess the function of all six suborgans of each labyrinth completely (145).

### Superior Vestibular Neuritis

The classic and most frequently encountered clinical pattern of UVL is that of superior VN (146, 147). These patients often present with an AVS and spontaneous horizontal–torsional nystagmus beating toward the healthy side. The vHIT—applicable directly in the emergency room—reveals loss of lateral and AC function with sparing of the PC, making the diagnosis of superior VN. In addition to the classic horizontal catch-up saccade upon rotation of the head toward the affected side, D’Onofrio (148) also described an oblique upward catch-up saccade after head rotation toward the healthy side. This catch-up saccade is thought to be caused by an isolated activation of the remaining inferior SCC contralateral to the head rotation, which drives the eyes downward, resulting in an oblique upward catch-up saccade (148). This results from the obligatory shift in VOR axis that occurs with UVL (29). Loss of utricular function—as confirmed by oVEMP testing—may complete the clinical pattern. The constellation can be explained by the anatomy of the superior branch of the vestibular nerve, which innervates the lateral and superior canal, as well as the utricle. The susceptibility of the superior branch for VN may be explained by the tight bony canal surrounding the vestibular nerve (149). The pattern of damage with sparing of the PC may also explain the propensity of these patients to benign paroxysmal positional vertigo. Weeks or months after the attack, debris from the affected canals may fall into the PC, which is still fully functional, and may cause recurrent attacks of positional vertigo.

### Inferior Vestibular Neuritis

The clinical counterpart of superior VN is inferior VN (146, 150). Here, only the PC is affected, often associated with tinnitus and hearing loss and saccular deficits measured with cVEMPs (146, 150–152). As there is little spontaneous nystagmus and normal caloric response, these do not fulfill the criteria of classic VN and may be mistaken as a central vestibular disorder. In these cases, detection of PC loss in vHIT may help differentiating between a peripheral and central vestibular disease.

### Bilateral Vestibular Loss with Anterior SCC Sparing

**Figure 15:**
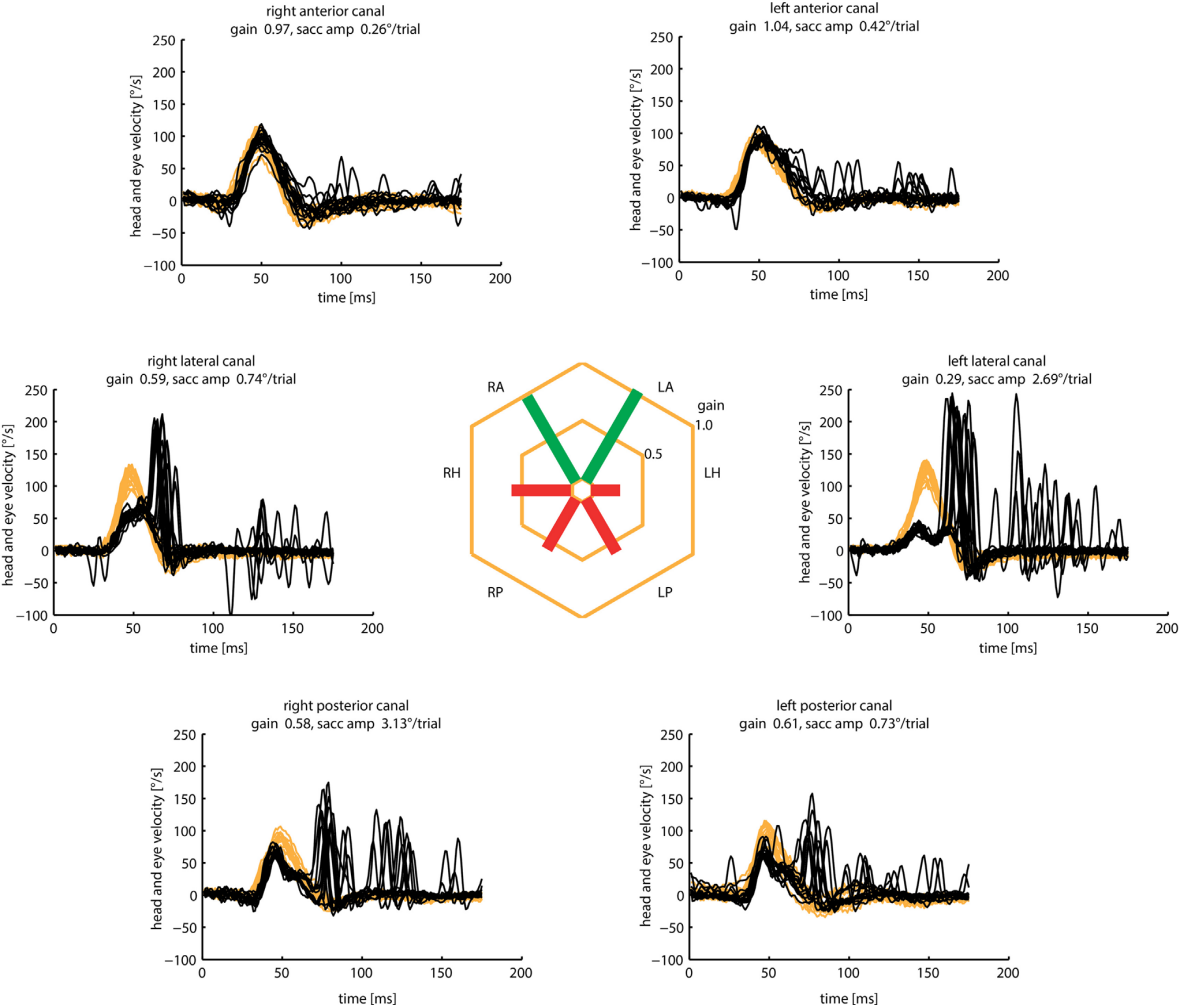
Bilateral vestibular loss (BVL) with anterior canal sparing. Video head impulse measures of patients with BVL often reveal relative sparing of anterior semicircular canal function. This clinical pattern may possibly indicate gentamicin vestibulotoxicity or Menière’s disease, but also occurs in BVL of unknown origin as in this patient.

Outside specialized balance centers, the diagnosis of BVL is often delayed or missed (153), because the characteristic symptoms of postural imbalance and oscillopsia (154–156)—often without vertigo—are not recognized. Once the suspicion has been raised, the clinical diagnosis is often straightforward based on three simple clinical bedside tests: the HIT, dynamic visual acuity, and the Romberg test on rubber foam (157). The etiology of BVL, however, still remains undetermined in about 50% of the cases despite intensive investigations (158). With the advent of vHIT, it is now possible to routinely assess the function of all six SCCs independently (4, 5, 9). Based on these investigations, characteristic patterns of SCC hypofunction started to emerge in patients with BVL: anterior SCC function was selectively spared in about 60% of these patients (Figure 15). As it turned out, AC sparing, determined by gain values (>0.7) and cumulative catch-up saccade amplitudes (<0.73°/trial), were associated with aminoglycoside vestibulotoxicity and Menière’s disease (17). Therefore, routine vHIT measures of all six SCCs may help to identify the underlying cause in patients with BVL.

### Unilateral and Bilateral Loss of PC Function

Isolated loss of posterior SCC function was found in about 2% of patients with vHIT assessment of all six SCCs (159). Thorough vestibular work-up of these patients, including oVEMP and cVEMP for testing otolith function, as well as audiograms to assess cochlear function, revealed associated vestibular deficits in more than 80% of these patients. These associated deficits were most frequent in patients with vestibular Schwannoma or a history of VN and often the patients were at risk for concomitant BPPV.

## Author Contributions

IC wrote Sections “Introduction,” “From Head Impulses to SHIMPs,” and “The Physiological Basis of the Head Impulse Test,” LM wrote Section “Practical Aspects and Potential Pitfalls of Video Head Impulse Testing,” HM wrote Section “Calculating VOR Gain,” LC and GH wrote Section “Quantitative Head Impulse Test in Central Vestibular Disorders,” and KW wrote Section “New Clinical Disease Patterns Emerging from 3D Video Head Impulse Testing.” GH and IC assembled the paper, and all authors revised it.

## Conflict of Interest Statement

IC, GH, HM, and LM are unpaid consultants to GN Otometrics, Taastrup, Denmark, but have received support from GN Otometrics for travel and attendance at conferences and workshops. For all other authors, the research was conducted in the absence of any commercial or financial relationships that could be construed as a potential conflict of interest.

## Abbreviations

AC, anterior canal; AICA, anterior inferior cerebellar artery; AT, ataxia telangiectasia; ATD, ascending tract of Deiter’s; AVS, acute vestibular syndrome; aWE, acute Wernicke’s encephalopathy; BVL, bilateral vestibular loss; CS-DI, compensatory saccade dysconjugacy index; GD, Gaucher’s disease; HC, horizontal canal; HIMP, the generic name for the head impulse test protocol; HIT, head impulse test; INO, internuclear ophthalmoplegia; ION, inferior olivary nucleus; LARP, the plane of the left anterior–right posterior canals; MLF, medial longitudinal fasciculus; NPH, nucleus prepositus hypoglossi; PC, posterior canal; PCS, posterior circulation stroke; PICA, posterior inferior cerebellar artery; RALP, the plane of the right anterior–left posterior canals; SCA 6, spinocerebellar ataxia type 6; SCC, semicircular canal; SHIMP, the generic name for the suppression head impulse test protocol; vHIT, video head impulse test; VN, vestibular neuritis; VOG, video-oculography; VOR, vestibulo-ocular reflex; VOR-DI, VOR dysconjugacy index.
